# MACHETE identifies interferon-encompassing chromosome 9p21.3 deletions as mediators of immune evasion and metastasis

**DOI:** 10.1038/s43018-022-00443-5

**Published:** 2022-11-07

**Authors:** Francisco M. Barriga, Kaloyan M. Tsanov, Yu-Jui Ho, Noor Sohail, Amy Zhang, Timour Baslan, Alexandra N. Wuest, Isabella Del Priore, Brigita Meškauskaitė, Geulah Livshits, Direna Alonso-Curbelo, Janelle Simon, Almudena Chaves-Perez, Dafna Bar-Sagi, Christine A. Iacobuzio-Donahue, Faiyaz Notta, Ronan Chaligne, Roshan Sharma, Dana Pe’er, Scott W. Lowe

**Affiliations:** 1 Cancer Biology & Genetics Program, Memorial Sloan Kettering Cancer Center, New York, NY USA; 2 Program for Computational and Systems Biology, Sloan Kettering Institute, Memorial Sloan Kettering Cancer Center, New York, NY USA; 3 PanCuRx Translational Research Initiative, Ontario Institute for Cancer Research, Toronto, Ontario Canada; 4 Louis V. Gerstner Jr. Graduate School of Biomedical Sciences, New York, NY USA; 5 Department of Biochemistry, New York University School of Medicine, New York, NY USA; 6 David M. Rubenstein Center for Pancreatic Cancer Research, Memorial Sloan Kettering Cancer Center, New York, NY USA; 7 Department of Pathology, Memorial Sloan Kettering Cancer Center, New York, NY USA; 8 Division of Research, Princess Margaret Cancer Centre, University Health Network, Toronto, Ontario Canada; 9 Department of Medical Biophysics, University of Toronto, Toronto, Ontario Canada; 10 Howard Hughes Medical Institute, Chevy Chase, MD USA

**Keywords:** Tumour immunology, Gene targeting, Cancer

## Abstract

The most prominent homozygous deletions in cancer affect chromosome 9p21.3 and eliminate *CDKN2A/B* tumor suppressors, disabling a cell-intrinsic barrier to tumorigenesis. Half of 9p21.3 deletions, however, also encompass a type I interferon (IFN) gene cluster; the consequences of this co-deletion remain unexplored. To functionally dissect 9p21.3 and other large genomic deletions, we developed a flexible deletion engineering strategy, MACHETE (molecular alteration of chromosomes with engineered tandem elements). Applying MACHETE to a syngeneic mouse model of pancreatic cancer, we found that co-deletion of the IFN cluster promoted immune evasion, metastasis and immunotherapy resistance. Mechanistically, IFN co-deletion disrupted type I IFN signaling in the tumor microenvironment, leading to marked changes in infiltrating immune cells and escape from CD8^+^ T-cell surveillance, effects largely driven by the poorly understood interferon epsilon. These results reveal a chromosomal deletion that disables both cell-intrinsic and cell-extrinsic tumor suppression and provide a framework for interrogating large deletions in cancer and beyond.

## Abstract (web summary)

Barriga et al. develop MACHETE, a genome engineering strategy enabling the flexible modeling of megabase-sized deletions and show that the concomitant loss of the interferon cluster with *CDKN2A/B* deletions on 9p21.3 enhances immune evasion.

## Main

Understanding the genetic underpinnings of cancer is a fundamental goal of cancer research. Most efforts have focused on the characterization of single-nucleotide variants (SNVs), which typically act as ON/OFF switches that affect the output of a single gene. An even larger class of cancer-associated lesions are copy-number alterations (CNAs), which alter the dosage of multiple linked genes^1,2^. Tumors have on average 24 distinct CNAs that impact up to 30% of the genome^3–5^. CNAs show recurrent patterns associated with clinical outcomes^3,4,6,7^, arguing for selection of specific biological traits rather than stochastic accumulation of genomic alterations. CNA research has commonly focused on known drivers within the affected regions, yet co-gained or co-deleted genes—once considered ‘passenger’ events—can contribute to tumorigenesis^1,8,9^. These observations imply that CNAs produce complex phenotypes that cannot be recapitulated by manipulating single genes^10–13^; however, modeling CNAs remains a major challenge that has impeded their functional assessment^11,12,14–16^.

Among recurrent CNAs, loss of chromosome 9p21.3 is the most strongly linked to poor prognosis as well as being the most common homozygous deletion across human cancers^3,6^. The 9p21.3 locus encompasses multiple key tumor-suppressor genes (TSGs): the cell cycle inhibitors *CDKN2A* (encoding p16^INK4a^ and p14^ARF^) and *CDKN2B* (encoding p15^INK4b^), which collectively activate the major tumor-suppressive pathways p53 and RB^17–19^. Hence, the current paradigm is that 9p21.3 deletions contribute to tumorigenesis by eliminating a proliferative block, yet several observations deviate from this model. Tumors with 9p21.3 deletions can display altered immune infiltrates^20,21^ and increased resistance to immune-checkpoint blockade (ICB)^22,23^, suggesting that the locus may also influence immune-related processes. Consistent with this notion, genome-wide association studies have identified SNVs in 9p21.3 even in non-cancer pathologies, notably age- and inflammation-related conditions^24^; however, the biological and molecular basis for these observations remains poorly understood.

For the functional study of deletions, CRISPR-Cas9 has been used to engineer these events^16,25^, yet standard approaches have low efficiency and thus require the isolation and screening of many clonal cell populations. Here, we developed a rapid and flexible approach to engineer megabase-sized deletions. We applied this approach to investigating 9p21.3 deletions in models of pancreatic cancer and melanoma.

## Results

### MACHETE enables efficient generation of megabase deletions

**Fig. 1:**
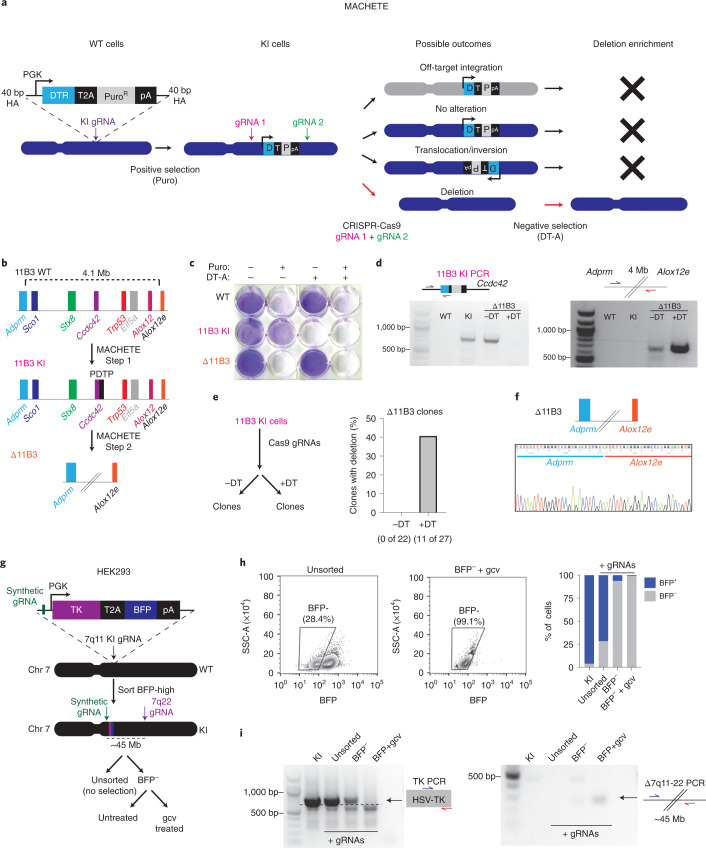
MACHETE enables efficient engineering of genomic deletions. **a**, Schematic of the MACHETE approach. WT, wild type; HA, homology arm; Puro, puromycin; pA, polyadenylation signal. **b**, Schematic of MACHETE-mediated engineering of a 4.1-Mb deletion at the 11B3 locus. **c**, Crystal violet stain of WT, 11B3 KI and Δ11B3 NIH3T3 cells after selection with Puro at 2 μg ml^−1^ and/or DT-A at 50 ng ml^−1^. **d**, PCR genotyping for the 11B3 KI and Δ11B3 alleles in the indicated NIH3T3 cell lines. **e**, Experimental outline for testing the impact of DT-mediated negative selection on the efficiency of Δ11B3 deletion engineering in NIH3T3 cells (left). Clonal analysis of NIH3T3 cells engineered without (−DT) and with (+DT) DT selection (right). **f**, Sanger sequencing of the 11B3 deletion breakpoint confirming the expected deletion. **g**, Schematic of MACHETE-mediated engineering of a 45-Mb deletion at the 7q11-22 locus in HEK293 cells. gcv, ganciclovir. **h**, Flow cytometry plots and quantification of BFP^+^ and BFP^−^ HEK293 cells under the indicated conditions. **i**, PCR genotyping for the 7q11 KI and Δ7q11-22 alleles in HEK293 cells under the indicated conditions. Source data

**Extended Data Fig. 1:**
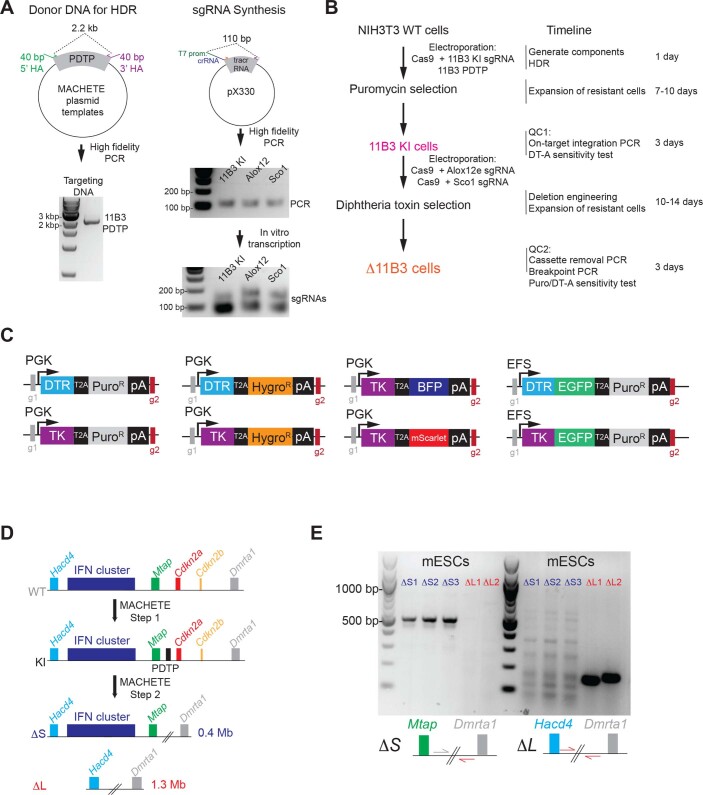
MACHETE allows rapid and flexible deletion engineering. (A) Preparation of donor DNA and sgRNA used for MACHETE-mediated targeting of the 11B3 locus in NIH3T3 cells. (B) Experimental outline and timing for MACHETE-based 11B3 deletion engineering in NIH3T3 cells. (C) Suite of dual selection cassettes generated for the MACHETE approach. (D) Schematic of MACHETE-mediated engineering of 4C4 deletions in mouse embryonic stem cells (mESCs). (E) PCR genotyping for the chromosome 4C4 ΔS and ΔL alleles in mESCs. Source data

To facilitate the experimental study of genomic deletions, we developed molecular alteration of chromosomes with engineered tandem elements (MACHETE) (Fig. 1a). First, a cassette encoding tandem negative and positive selection markers is amplified and inserted into the region of interest by CRISPR-facilitated homology-directed repair, then cells with integrations are enriched by positive selection. Second, a pair of single guide RNAs (sgRNAs) targeting the breakpoints of the intended deletion are introduced, which is followed by negative selection. The sequence specificity of the flanking guides exclusively deletes on-target integrations of the suicide cassette, thereby eliminating cells that either retain the selection cassette or have off-target integrations (Fig. 1a). The MACHETE protocol was designed to eliminate the need for cloning components: donor DNA is generated by introducing 40-bp homology arms via PCR amplification of the selection cassette, which is coupled to ribonucleoproteins (RNPs) of Cas9 with sgRNAs (Extended Data Fig. 1a,b).

As proof of concept, we engineered a 4.1-Mb deletion of the murine 11B3 locus (syntenic to human 17p13.1), which encompasses the *Trp53* TSG (Fig. 1b) and had been previously engineered using a Cre/loxP approach^12^. NIH3T3 fibroblasts were targeted with a PGK promoter–driven diphtheria toxin receptor linked to puromycin resistance by a self-cleaving 2A peptide (PGK-DTR–T2A–Puro (PDTP)) dual-selection cassette to an intronic region of *Ccdc42*, a gene located in the 11B3 locus and selected for insertion of the cassette (11B3 knock-in (KI) cells). Cas9-sgRNA RNPs were then introduced to target regions flanking *Sco1* and *Alox12*, the genes that demarcate the intended deletion, and negative selection was performed using diphtheria toxin (DT) to produce Δ11B3 cells (Fig. 1b). Parental, 11B3 KI and Δ11B3 populations showed the expected pattern of resistance or sensitivity to the selection agents (Fig. 1c) and the expected presence/absence of the cassette and deletion breakpoint (Fig. 1d). Clonal analysis showed that DT selection effectively enabled the generation of the desired deletion, by increasing the frequency of Δ11B3 cells from undetectable (0 of 22) to 40% of positive clones (11 of 27, all heterozygous) (Fig. 1e), which was confirmed by sequencing (Fig. 1f). We also developed a series of constructs that expand the applicability of MACHETE (Extended Data Fig. 1c), which we applied to illustrate its use in human cells. We tested one of these constructs (HSV–TK–T2A–BFP), which allowed the generation of cells harboring a 45-Mb deletion of chromosome 7q11–7q22 (Fig. 1g–i). Finally, to demonstrate the utility of MACHETE beyond cancer cells, we engineered 0.4 and 1.3-Mb deletions of chromosome 4C4 in mouse embryonic stem cells, thus enabling the creation of germline deletion events (Extended Data Fig. 1d,e). Hence, MACHETE is a customizable approach to efficiently engineer large chromosomal deletions across a variety of cellular systems.

### Loss of type I IFN genes in 9p21.3 deleted tumors

**Fig. 2:**
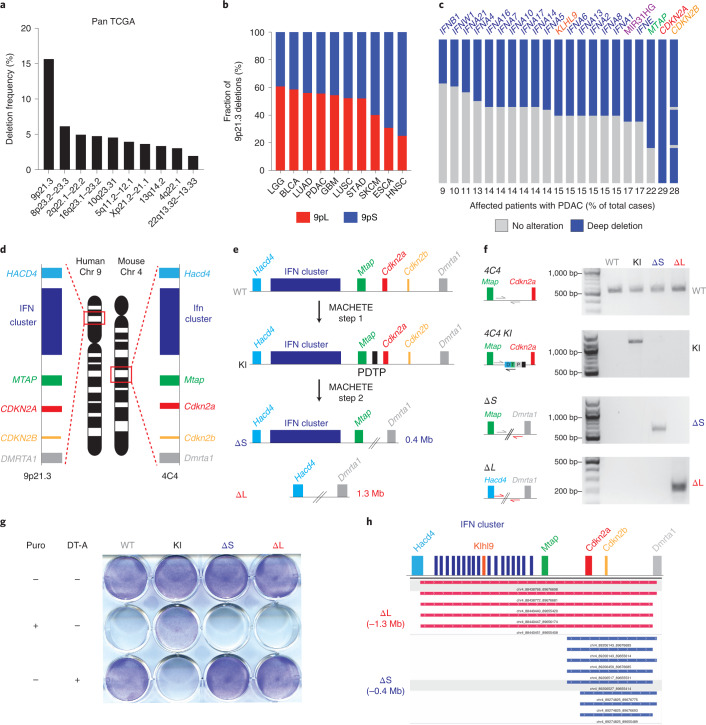
9p21.3 deletions encompass a cluster of type I IFNs. **a**, Frequency of homozygous deletions across the pan-cancer TCGA dataset. **b**, Relative frequency of deletions at the 9p21.3 locus classified as 9pS and 9pL across different cancer types. **c**, Frequency of deep deletion of 9p21.3 genes in patients with PDAC. **d**, Schematic of the synteny between the human 9p21.3 and mouse 4C4 locus. **e**, Schematic of MACHETE-mediated engineering of 4C4 ΔS and ΔL deletions. **f**, PCR genotyping for the WT, KI, ΔS and ΔL alleles in the indicated PDEC cell lines. **g**, Pattern of resistance/sensitivity to positive and negative selection in PDEC sgP53 EL parental, 4C4 KI, ΔS and ΔL cells. Cells were seeded and treated with puromycin (2 μg ml^−1^) or DT-A (50 ng ml^−1^) for 72 h and then stained with crystal violet to assess surviving cells. **h**, DNA sequencing of breakpoints from ΔS and ΔL cells confirming loss of the expected genomic regions (0.4 Mb deletion in ΔS and 1.3 Mb deletion in ΔL). Source data

**Extended Data Fig. 2:**
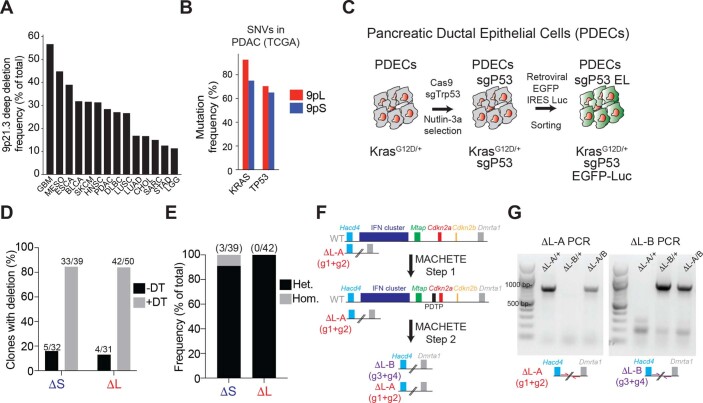
Generation of 4C4 deletions in PDECs. (A) Frequency of deep deletions at the 9p21.3 locus across different types of cancer in the TCGA dataset. (B) Mutation frequency of *KRAS* and *TP53* in 9pL and 9pS PDACs in the TCGA dataset. (C) Schematic of the generation of PDEC sgP53 EL cells. CRISPR-mediated knockout of *Trp53* was done by electroporation of a pX330-sgP53 plasmid followed by treatment with Nutlin-3 (10 μM) to select for *Trp53*-deficient cells. PDEC sgP53 cells were then infected with a retroviral EGFP-luciferase construct and cells were selected by sorting for EGFP + expression. (D) Clonal analysis of ΔS and ΔL cells engineered without (-DT) and with (+DT) diphtheria toxin selection. (E) Frequency of heterozygous and homozygous ΔS or ΔL deletions in PDEC cells following MACHETE engineering. (F) Schematic of iterative editing of cells bearing a heterozygous ΔL deletion, using a distinct set of guides to discern between the different deletions. (G) PCR genotyping of the distinct ΔL deletion breakpoints. Source data

Armed with MACHETE, we set out to interrogate 9p21.3 deletions (Fig. 2a). These deletions almost invariably affect the tumor-suppressor gene *CDKN2A;* however, we and others have noted that 9p21.3 deletions can encompass additional genes, including a cluster of 16 type I IFNs. Loss of these IFN genes has not been functionally implicated in tumorigenesis, despite the known role of IFN signaling in antitumor immunity^26^. An analysis of the TCGA dataset^27^ revealed that 14 different tumor types harbor homozygous 9p21.3 deletions in over 10% of cases (Extended Data Fig. 2a). We further classified 9p21.3 deletions into those targeting *CDKN2A/B* alone (9p small or 9pS) or larger events that typically encompassed the entire type I IFN cluster (9p large or 9pL) (Fig. 2b). The frequency of the 9pL events ranged between 20–60% depending on tumor type and was among the highest in pancreatic ductal adenocarcinoma (PDAC) (Fig. 2c).

### Engineering 9p21.3 deletions in mouse models of PDAC

Genetic analyses of human PDAC indicate that *CDKN2A* deletions are an early event in tumor evolution^28,29^. They are thought to emerge as heterozygous deletions that subsequently undergo loss of heterozygosity^30,31^. These deletions tend to co-occur with activating *KRAS* mutations and *TP53* loss, two other major drivers in this disease (Extended Data Fig. 2b)^32^. Given the role of type I IFNs in modulating immunity, we set out to study 9p deletions in a syngeneic model of murine PDAC derived from established pancreatic ductal epithelial cells (PDECs) that harbor an endogenous *Kras*^*G12D*^ allele^33,34^. The lesions produced following PDEC transplantation resemble premalignant stages of PDAC, with limited capacity to progress to adenocarcinoma^34^ and the model allows the study of immune-related processes^33,35^. Given the synteny between human 9p21.3 and murine 4C4 (Fig. 2d), PDECs provide a suitable platform for MACHETE-based engineering of 9p21.3-equivalent deletions in vitro and the subsequent study of tumor phenotypes in an immune-competent context.

We generated *Trp53* knockout PDEC cells using transient CRISPR-Cas9 and introduced an EGFP-luciferase cassette to enable visualization of engrafted cells (PDEC sgP53-EL cells) (Extended Data Fig. 2c). MACHETE was then used to engineer the two most frequent configurations of 9p21.3 deletions: ΔS (‘small’; 0.4-Mb loss spanning *Cdkn2a* and *Cdkn2b*) and ΔL (‘large’; 1.3-Mb loss spanning the entire 4C4 locus) (Fig. 2e–g). Breakpoint sequencing confirmed the presence of precise 0.4- and 1.3-Mb deletions (Fig. 2h) and clonal analysis of targeted cell populations indicated that MACHETE achieved more than an eightfold increase in producing cells with the intended heterozygous deletion (Extended Data Fig. 2d,e). As expected, these populations could be further edited through MACHETE’s capability for iterative engineering (Extended Data Fig. 2f,g). Given the comparable deletion efficiency of ΔS and ΔL cells, we used cell populations rather than individual clones for subsequent analyses to minimize the effects of clonal variation.

### ΔL tumors are differentially surveilled by the immune system

**Fig. 3:**
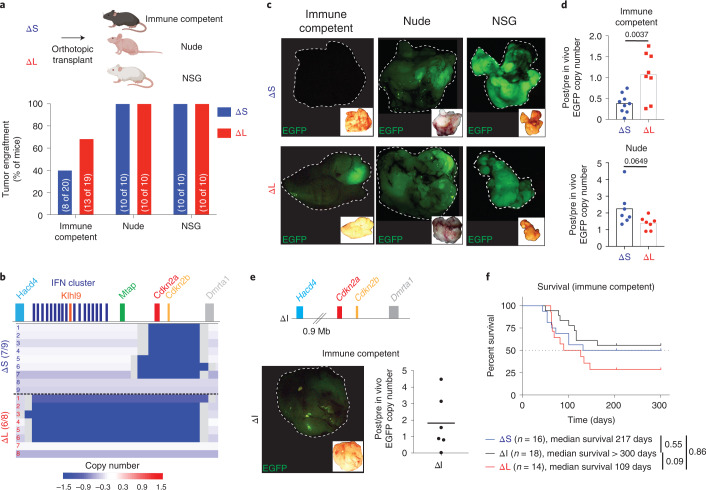
Large 4C4 deletions evade immunoediting. **a**, Tumor engraftment at 1 month after injection of ΔS and ΔL cells in C57BL/6, nude and NSG hosts. Two independently generated input cell lines were used per genotype. Bars represent fraction of tumor-bearing mice (specific numbers of independently analyzed mice are noted in parentheses). **b**, Sparse whole-genome sequencing analysis of 4C4 deletion status in ΔS and ΔL tumor-derived cell lines (from C57BL/6 hosts). Each row is an independent tumor-derived line (ΔS, *n* = 9; ΔL, *n* = 8). Deep blue color depicts deletion defined as log_2_ relative abundance <−2. **c**, Representative macroscopic fluorescent images of primary tumors collected from the indicated genotypes and hosts (Images were taken from ten mice per genotype per host). Insets show the brightfield image for each tumor. **d**, qPCR analysis for EGFP copy number in the genomic DNA of tumor-derived (post in vivo) ΔS and ΔL lines from C57BL/6 and nude hosts, relative to their parental (pre in vivo) counterparts. Each dot represents an independent tumor-derived cell line (*n* = 8 C57BL/6, 7 nude; for ΔS and ΔL). Groups were compared using a two-tailed unpaired Student’s *t*-test. **e**, Schematic representation of the MACHETE-engineered ΔI allele that removes an 0.9-Mb region between *Hacd4* and *Cdkn2a* (top). Representative macroscopic image of a ΔI tumor showing retained EGFP expression at end point (bottom left). Inset shows matched brightfield image. qPCR analysis for EGFP copy number in the genomic DNA of tumor-derived (post in vivo) ΔI cell lines from C57BL/6 hosts relative to their parental (pre in vivo) counterparts (bottom right). Each dot represents an independent cell line (*n* = 6). **f**, Survival curve of C57BL/6 mice transplanted with ΔS, ΔI, or ΔL tumor cells. Curves were compared using a log-rank test. Source data

**Extended Data Fig. 3:**
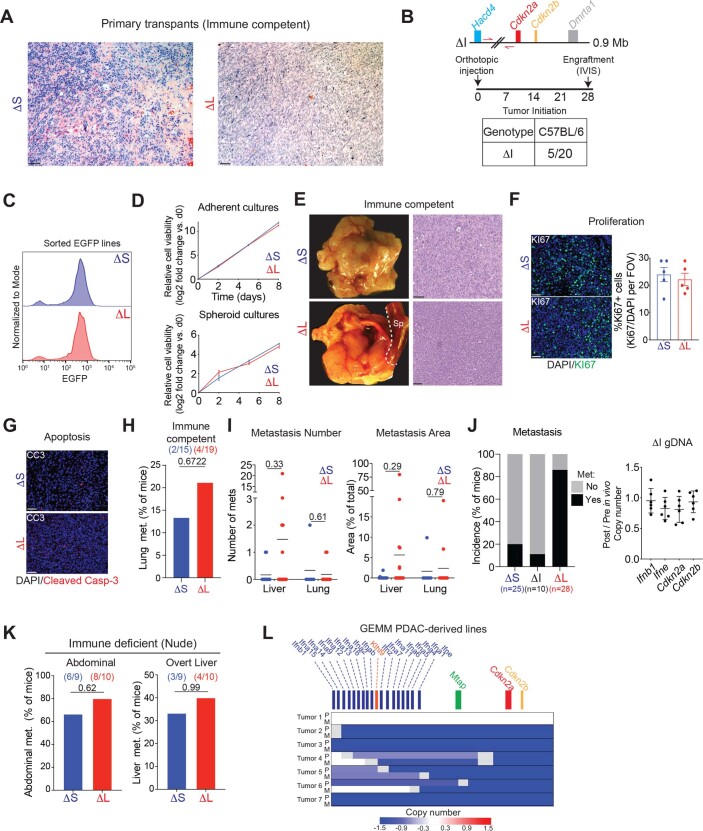
Large 4C4 deletions promote immune evasion and metastasis in a host-dependent manner. (A) Histology of ΔS and ΔL tumors in C57BL/6 mice. Representative H/E images are shown. Scale bar is 100 μm. (B) (Top) Schematic representation of the MACHETE-engineered ΔI allele that removes a 0.9 Mb region between *Hacd4* and *Cdkn2a*. (Bottom) Engraftment of ΔI cells in C57BL/6 mice one month after injection, measured by bioluminescence. (C) EGFP levels of representative re-sorted tumor-derived ΔS and ΔL cell lines (repeated 4 times). (D) Growth curves in adherent (top) or suspension (bottom) conditions for ΔS and ΔL cell lines. (E) Macroscopic images (left) and hematoxylin/eosin stain (right) of orthotopic tumors in C57BL/6 mice transplanted with tumor-derived ΔS and ΔL cells. Scale bar is 200 μm. (F) Representative images (left) and quantification (middle) of the fraction of Ki67+ cells in ΔS and ΔL tumors. Scale bar is 100 μm. Each dot represents an independent biological replicate (n = 5). (G) Representative images of cleaved caspase-3 in ΔS and ΔL tumors, which showed little to no detectable signal. Scale bar is 100 μm. (H) Lung metastasis incidence in C57BL/6 mice with either ΔS or ΔL tumors. Bars represent the fraction of metastasis-bearing mice. Differences were assessed by Fisher’s exact test. Each dot represents an independent biological replicate (ΔS n = 17, ΔL n = 23). (I) Quantification of the number (left) and relative area (right) of liver and lung metastases in C57BL/6 mice with either ΔS or ΔL tumors. Each dot represents an independent biological replicate (Liver ΔS n = 12, ΔL n = 21; Lung ΔS n = 6, ΔL n = 11). Differences were assessed by two-tailed unpaired test. (J) (Left) Metastasis incidence in C57BL/6 mice with ΔS, ΔI, or ΔL tumors. (Right) Copy number of *Ifnb1*, *Ifne*, *Cdkn2a*, and *Cdkn2b* in tumor-derived ΔI lines (Post) relative to pre-injection parental ΔI cells (Pre). Each dot represents an independent tumor-derived line (n = 5). ΔS and ΔL data are also used in Fig. 4. (K) Metastasis incidence in Nude mice with either ΔS or ΔL tumors. Each dot represents an independent biological replicate (Abdominal ΔS n = 9, ΔL n = 10; Liver ΔS n = 9, ΔL n = 10). (L) Analysis of 4C4 deletion status in PDAC GEMM cell lines derived from matched primary tumors (‘P’) and metastases (‘M’). Each row is paired primary and metastasis-derived cell lines (n = 7 pairs). Sparse WGS was used to assess the status of the 4C4 locus. Deep blue color depicts deletion, defined as log2 relative abundance < −2. Source data

To assess the role of 4C4 heterozygous deletions in tumorigenesis, we transplanted the ΔS and ΔL lines into the pancreata of syngeneic C57BL/6 recipients. Cells bearing the ΔL deletion tended to form more tumors than ΔS cells, although the difference was not statistically significant (Fig. 3a). Tumors arising from both genotypes were poorly differentiated (Extended Data Fig. 3a), consistent with the histopathology of autochthonous *Trp53*- and *Cdkn2a*-deficient PDAC models^36^. Sparse whole-genome sequencing confirmed that most ΔS and ΔL tumors acquired homozygous deletions of their respective alleles (seven of nine lines for ΔS; six of eight lines for ΔL), as occurs in human PDAC (Fig. 3b).

We found one notable difference between ΔL and ΔS tumors; ΔL tumors retained a stronger EGFP fluorescence signal and genomic copy number (Fig. 3c,d). These observations are consistent with immunoediting of cells with high reporter expression^37^ and raise the possibility that ΔL cells may be less immunogenic than their ΔS counterparts. Accordingly, ΔS and ΔL cells showed a similar capability of forming EGFP-expressing tumors in *Foxn1*^nu^ (‘nude’: T and B-cell-deficient) and NOD/SCID *Il2rg*^−/−^ (NSG: T, B and natural killer (NK) cell-deficient) mice (Fig. 3a,c,d). We examined a 4C4 deletion (ΔI allele) that retains the *Cdkn2a/b* genes but deletes the IFN cluster and adjacent genes. ΔI cell populations had reduced tumor initiating capacity, yet produced EGFP-positive tumors comparable to the ΔL allele (Fig. 3e,f and Extended Data Fig. 3b). These data indicate that the genomic region upstream of *Cdkn2a/b* contributes specifically to tumor immunoediting.

### ΔL deletions promote metastasis by evading adaptive immunity

**Fig. 4:**
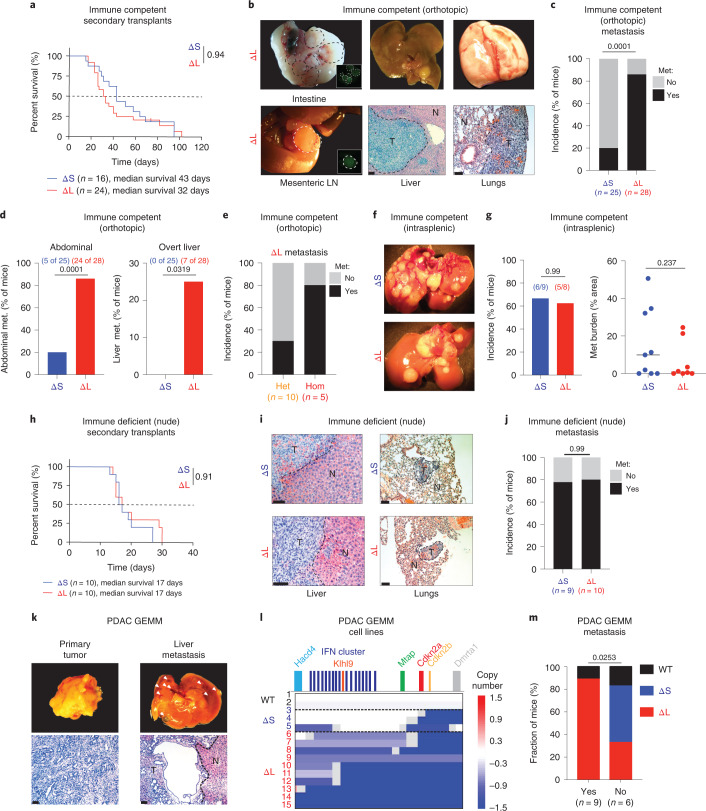
Large 4C4 deletions promote metastasis. **a**, Survival curve of C57BL/6 mice transplanted with tumor-derived ΔS and ΔL cells. Curves were compared using a log-rank test. **b**, Representative images of metastases in C57BL/6 mice with ΔL tumors. Brightfield macroscopic images of abdominal (intestinal and mesenteric lymph node (LN)) metastases (met.) (left). Insets show matched EGFP fluorescence images. Macroscopic and hematoxylin & eosin images of tumor-bearing livers (middle). Scale bar, 200 μm. Macroscopic and hematoxylin & eosin images of tumor-bearing lungs (right). Scale bar, 100 μm. T, tumor; N, normal adjacent tissue. **c**–**e**, Overall (**c**), organ-specific (**d**) and zygosity-specific (**e**) metastasis incidence in C57BL/6 mice. Two to four independently generated input cell lines were used per genotype. Bars represent fraction of metastasis-bearing mice (independently analyzed mice are noted in parentheses) and differences were assessed with Fisher’s exact test. **f**, Macroscopic images of liver metastases in C57BL/6 mice after intrasplenic injection of either ΔS or ΔL cells. **g**, Incidence and relative area of liver metastases in C57BL/6 mice after intrasplenic injection of either ΔS or ΔL cells. Bars represent fraction of metastasis-bearing mice (independently analyzed mice are noted in parentheses) (left). Each dot represents an independent mouse (ΔS, *n* = 9; ΔL, *n* = 8) (right). Differences were assessed with Fisher’s exact test and burden with an unpaired two-tailed Student’s *t*-test. **h**, Survival curves of nude mice transplanted with tumor-derived ΔS or ΔL cells. Curves were compared using a log-rank test. **i**, Representative images of metastases in nude mice with ΔL or ΔS tumors. Hematoxylin/ eosin images of tumor-bearing livers (left) and lungs (right) are shown. Scale bar, 100 μm. **j**, Overall metastasis incidence in nude mice. Bars represent fraction of independent mice with metastasis, ΔS (*n* = 9) or ΔL (*n* = 10) tumors. Differences were assessed with Fisher’s exact test. **k**, Representative gross morphology (top) and hematoxylin & eosin histological stain (bottom) of matched primary tumor and overt liver metastasis in a Kras^G12D/+^; shSmad4 PDAC GEMM. Scale bar, 200 μm. **l**, Sparse WGS analysis of tumor-derived cell lines from the KC-Ren and KC-Smad4 GEMMs, grouped by spontaneous 4C4 deletion type (WT, ΔS and ΔL). Blue tracks indicate deleted regions, with color intensity corresponding to the extent of the deletion. Numbers correspond to independent mice. (M) Incidence of WT 4C4 locus, ΔS deletion or ΔL deletion among tumors with or without associated overt metastases. Specific numbers of independently analyzed mice are noted in parentheses. Differences were assessed with chi-squared test. Source data

Next, we compared the behavior of ΔS and ΔL tumor-derived cell lines in orthotopic transplantation assays. Four independently derived ΔS and ΔL tumor lines were FACS-sorted to obtain cell populations with comparable EGFP levels to eliminate differences in reporter expression as a confounding factor (Extended Data Fig. 3c). ΔS and ΔL tumor cells showed a similar ability to proliferate in adherent or suspension cultures and, upon transplantation, produced tumors with undifferentiated histopathology (Extended Data Fig. 3d,e). Consistent with their acquisition of homozygous 4C4 deletions, the tumors progressed more rapidly compared to tumors from the parental ΔS and ΔL cell pools (Fig. 4a,f).

Although ΔS and ΔL tumors showed no obvious difference in the fraction of proliferating or apoptotic cells (Extended Data Fig. 3f,g), ΔL tumors were more prone to metastasis (Fig. 4b,c), displaying a fourfold increase in macrometastases in the abdomen (in mesenteric lymph nodes, intestine and peritoneal cavity) compared to their ΔS counterparts and uniquely harbored overt liver metastases (~25% of mice) (Fig. 4d). These observations were confirmed through histological analyses, which also indicated that ΔL tumors tended to give rise to a larger number and area of liver lesions (Extended Data Fig. 3h,i).

To gain further mechanistic insights, we used additional tumor genotypes, additional routes of cell delivery, and immunocompromised hosts. First, tumor-derived cells that remained heterozygous (that is, no loss of heterozygosity) for the ΔL or ΔI alleles were unable to efficiently produce metastases following orthotopic injection (Fig. 4e and Extended Data Fig. 3j). Second, homozygous ΔS or ΔL tumor cells had comparable metastatic capacity following intrasplenic injection (Fig. 4f,g). Third, homozygous ΔS and ΔL cells showed similar rates of metastasis in nude mice (Fig. 4h–j and Extended Data Fig. 3k). Therefore, the enhanced metastatic propensity of ΔL cells requires concomitant homozygous deletion of *Cdkn2a/b* and the IFN cluster, and involves an immune surveillance mechanism that acts before colonization of distant sites.

We confirmed the association between spontaneous large 4C4 deletions and metastasis in autochthonous genetically engineered mouse model (GEMM) of PDAC which bears mutant *Kras*^*G12D*^ alone or in combination with *Smad4* depletion (Fig. 4k–m). Tumors in this model displayed a moderately differentiated histology with stromal involvement (Fig. 4k) and the presence and extent of 4C4 deletions was similar between individual primary and metastatic pairs (Extended Data Fig. 3l). These orthogonal data reinforce the notion that one or more genes that lie within the ΔL deletion but not the ΔS deletion suppress metastasis.

### ΔL deletions alter the response to immunotherapy

**Fig. 5:**
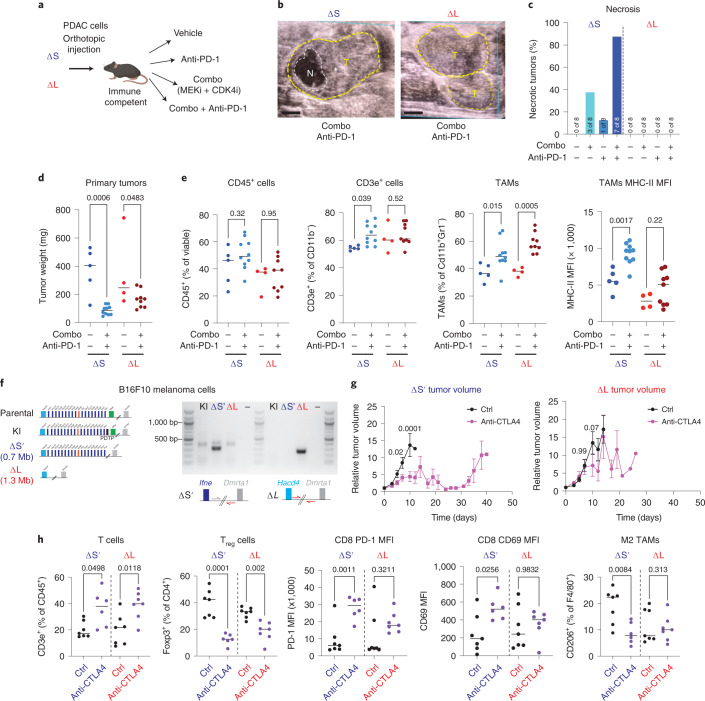
Loss of type I IFNs alters the response to ICB. **a**, Experimental setup to test the effects of ICB in ΔS and ΔL tumors. **b**, Representative ultrasound images of tumors (T, circled in yellow) with observed necrotic region (N, circled in white) (left). Scale bars, 1 mm. **c**, Frequency of necrosis in ΔS and ΔL tumors. (*n* = 8–9 independent mice per group). MFI, mean fluorescence intensity. **d**, ΔS and ΔL tumor weights 1 week after vehicle or combo + PD-1 treatment. Differences were assessed with one-way analysis of variance (ANOVA) followed by Sidak’s multiple comparison between vehicle and combo + PD-1-treated tumors (ΔS vehicle *n* = 5, ΔS treated *n* = 10, ΔL vehicle *n* = 4, ΔL treated *n* = 9 independent mice per condition). **e**, Frequency of CD45^+^ (far left), CD3e^+^ (left), TAMs (right) and surface expression of major histocompatibility complex (MHC)-II in TAMs (far right) in ΔS and ΔL tumors treated with vehicle or combo + PD-1. Differences were assessed with one-way ANOVA followed by Sidak’s multiple comparison between vehicle and combo + PD-1-treated tumors (ΔS vehicle *n* = 5, ΔS treated *n* = 10, ΔL vehicle *n* = 4, ΔL treated *n* = 9 independent mice per condition). **f**, Schematic of the ΔS′ and ΔL alleles engineered in B16F10 cells to test the response to anti-CTLA4 (left). Genotyping PCR of the expected breakpoints for the ΔS′ and ΔL alleles (right). **g**, ΔS′ (left) or ΔL (right) tumor volume after treatment with vehicle or anti-CTLA4 (*n* = 10 independent mice per group). Dots represent mean and bars represent s.e.m. Differences were assessed with two-way ANOVA followed by Sidak’s multiple comparison between vehicle and anti-CTLA4-treated tumors. **h**, Frequency of T cells (CD3e^+^CD11B^−^) (far left); frequency of Foxp3^+^ T regulatory cells (left); surface levels of PD-1 in CD8^+^ T cells (middle); surface levels of CD69 in CD8^+^ T cells (right); and frequency of CD206^+^ TAMs (far right) in ΔS′ and ΔL tumors treated for 1 week with vehicle or anti-CTLA4. Differences were assessed with one-way ANOVA followed by Sidak’s multiple comparison between vehicle and anti-CTLA4 (ΔS vehicle *n* = 7, ΔS-treated *n* = 6, ΔL vehicle *n* = 7 and ΔL-treated *n* = 7 independent mice per condition). Source data

**Extended Data Fig. 4:**
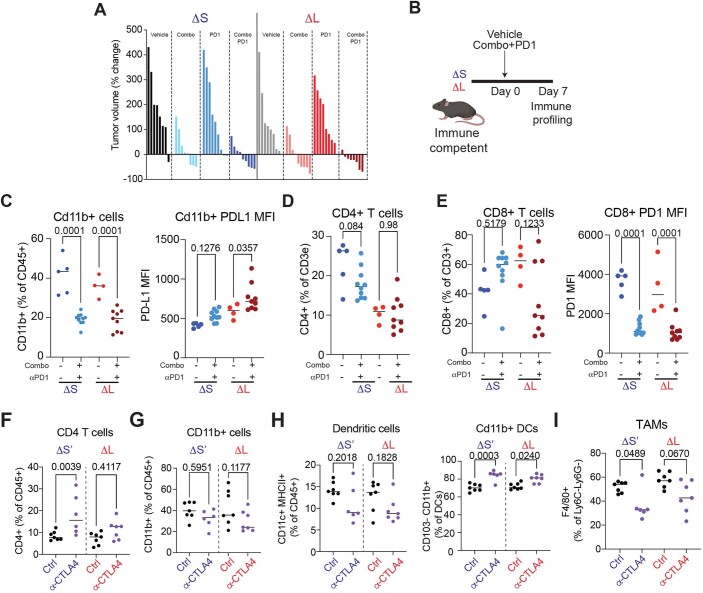
4C4 deletions dictate the response to immune checkpoint blockade. (A) Waterfall plot of percentage of tumor volume change. Tumors were measured at baseline and 14 days after treatment with vehicle, Combo (MEK and CDK4/6 inhibitors), α-PD1, or Combo + α-PD1. Each bar represents an independent biological replicate (n = 8-9 per group). (B) Experimental setup to assess immune infiltration in ΔS and ΔL tumors before onset of necrosis. (C) (Left) Frequency of CD11B + cells; (Right) Median PD-L1 surface expression of CD11B + cells in ΔS and ΔL tumors treated with vehicle or Combo+PD1. Differences were assessed Differences were assessed with one-way ANOVA followed by Sidak’s multiple comparison between vehicle and Combo+PD1 treated tumors (ΔS vehicle n = 5, ΔS treated n = 10, ΔL vehicle n = 4, ΔL treated n = 9 independent mice per condition). (D) Frequency of CD4 T cells (CD3e + CD4 + ) in ΔS and ΔL tumors treated with vehicle or Combo+PD1. Differences were assessed with one-way ANOVA followed by Sidak’s multiple comparison between vehicle and Combo+PD1 treated tumors (ΔS vehicle n = 5, ΔS treated n = 10, ΔL vehicle n = 4, ΔL treated n = 9 independent mice per condition). (E) (Left) Frequency of CD8 T cells (CD3e + CD8 + ); (Right) Median PD1 surface expression of CD8 T cells in ΔS and ΔL tumors treated with vehicle or Combo+PD1. Differences were assessed with one-way ANOVA followed by Sidak’s multiple comparison between vehicle and Combo+PD1 treated tumors (ΔS vehicle n = 5, ΔS treated n = 10, ΔL vehicle n = 4, ΔL treated n = 9 independent mice per condition). (F-I) Frequency of the indicated cell types in ΔS’ and ΔL tumors treated for one week with vehicle or α-CTLA4 condition). (F) CD4 T cells (CD3e + CD4 + ); (G) CD11B + cells; (H) (Left) Dendritic cells (CD11C + MHCII + ), (Right) CD11B + DCs (CD11C + MHCII + CD11B + CD103-); (I) TAMs (CD11B + CD11C- Ly6G- Ly6C- F4/80 + ). Differences were assessed with one-way ANOVA followed by Sidak’s multiple comparison between vehicle and α-CTLA4 (ΔS vehicle n = 7, ΔS treated n = 6, ΔL vehicle n = 7, ΔL treated n = 7 independent mice per condition). Source data

Chromosome 9p21.3 deletions portend poor outcome to ICB^22,23^, yet the molecular basis for this association has not been functionally established^38^. We evaluated response of ΔS and ΔL tumors to ICB combined with MEK and CDK4/6 inhibitors, which were previously shown to cooperate with anti-PD-1 in a model of PDAC^39^. Both genotypes responded to MEK + CDK4/6 inhibition (Fig. 5a–c and Extended Data Fig. 4a); however, ΔS tumors uniquely showed induction of necrosis detected by ultrasound (Fig. 5b,c), which was preceded by an early reduction in tumor size, increase in CD3e^+^ T-cell infiltration and engagement of an antitumor myeloid cell phenotype (Fig. 5d,e and Extended Data Fig. 4b–e). These data show that large 4C4 deletions can alter the responsiveness of PDAC to ICB.

We also performed analogous experiments in melanoma, where ICB is routinely used in the clinic. Starting with B16F10 cells (Fig. 5f), a murine model that responds to anti-CTLA4 immunotherapy^40^, we engineered two alleles: ΔL (deletion from *Hacd4* to *Dmrta1*) and ΔS′ (deletion spanning *Mtap* to *Dmrta1*). Following production of subcutaneous tumors in syngeneic hosts, mice were treated with anti-CTLA4 therapy and monitored for tumor response and changes in immune infiltration. In line with our PDAC findings, ΔS melanoma growth was impaired following anti-CTLA4 treatment, whereas ΔL tumors were largely refractory (Fig. 5g). Tumor immunophenotyping 1 week after treatment indicated loss of Foxp3^+^ regulatory T (T_reg_) cells in response to CTLA4 across genotypes (Fig. 5h and Extended Data Fig. 4f–i). Only ΔS′ tumors, however, exhibited increased activation of CD8^+^ T cells and loss of CD206^+^ tumor-associated macrophages, demonstrating that genes unique to ΔL deletions are required to elicit an effective antitumor response (Fig. 5h and Extended Data Fig. 4f–i). Overall, these data show that ΔL deletions promote resistance to ICB.

### 4C4 deletion genotype dictates type I IFN signaling and immune infiltration

**Fig. 6:**
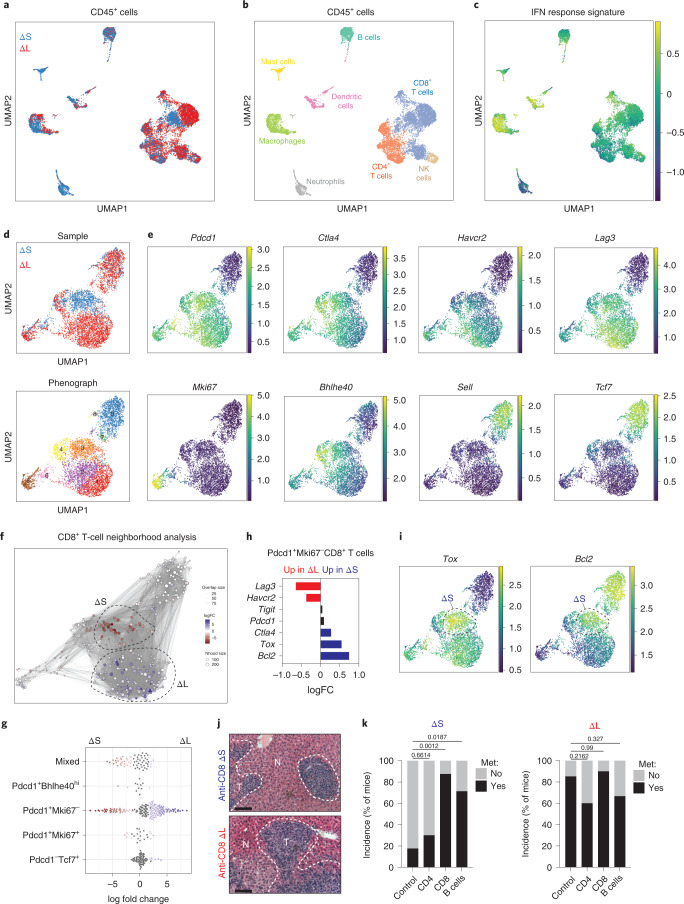
4C4/9p21.3 deletion genotype dictates type I IFN signaling and immune infiltration. **a**, UMAP of CD45^+^ cells showing cells derived from ΔS (*n* = 7,774 cells) or ΔL (*n* = 7,560 cells) tumors. **b**, UMAP of CD45^+^ cells annotating the specific immune subsets. **c**, UMAP of averaged IFN response signature across CD45^+^ populations. **d**, UMAP of CD8^+^ T cells from ΔS or ΔL tumors (top). Cells are colored by sample. UMAP of CD8^+^ T-cell clusters (bottom). Cells are colored by their cluster identity. **e**, UMAP of imputed expression for the indicated genes. **f**, Milo analysis of CD8^+^ T cells. Neighborhoods identified through Milo analysis using default parameters (red indicates enrichment in ΔS and blue indicates enrichment in ΔL). FC, fold change. **g**, Swarm plot of the distribution of CD8^+^ T-cell neighborhoods in ΔS or ΔL tumors across transcriptional states. The *x* axis indicates the log_2_(fold change) in differential abundance of ΔS (<0) and ΔL (>0). Each neighborhood is associated with a cell type if more than 80% of the cell state in the neighborhood belong to said state, else it is annotated as ‘mixed’. **h**, Differential gene expression of the indicated genes in *Pdcd1*^+^*Mki67*^*−*^CD8^+^ T cells. **i**, UMAP of imputed expression of *Tox* and *Bcl2*. Dashed circles highlight ΔS-enriched CD8^+^ T cells. **j**, Representative images of liver metastasis upon CD8^+^ cell depletion. Scale bar, 100 μm. **k**, Incidence of metastasis upon depletion of immune subsets in ΔS or ΔL tumors. Two independently generated input cell lines were used per genotype. Bars represent fraction of metastasis-bearing mice (ΔS control *n* = 25, ΔS anti-CD4 *n* = 10, ΔS anti-CD8 *n* = 8, ΔS anti-CD20 *n* = 7, ΔL control *n* = 28, ΔL anti-CD4 *n* = 5, ΔL anti-CD8 *n* = 10 and ΔL anti-CD20 *n* = 9 independent mice per condition). Differences were assessed with Fisher’s exact test. Source data

**Extended Data Fig. 5:**
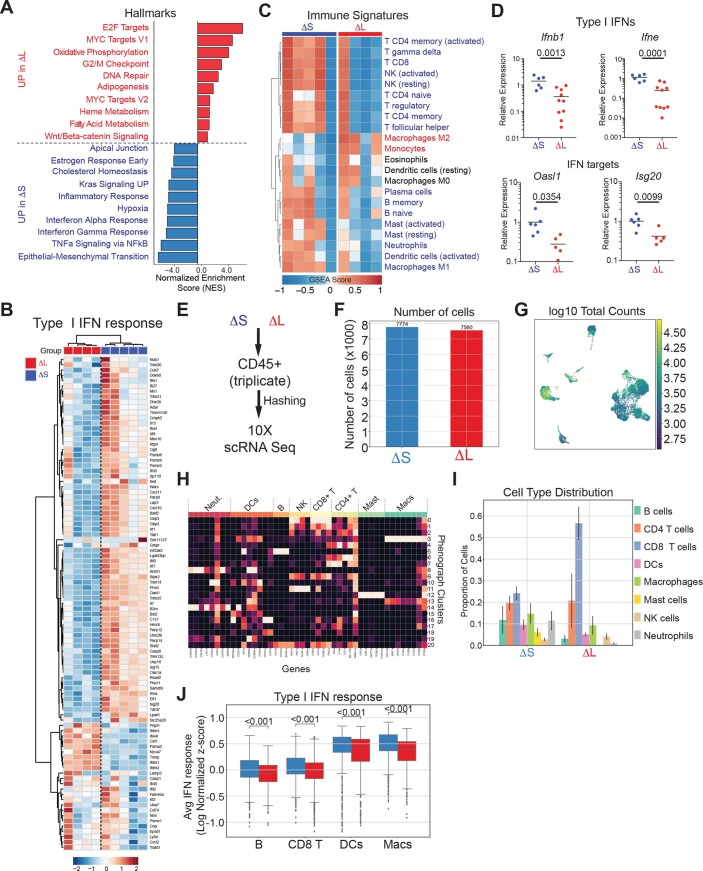
4C4 deletions determine the levels of IFNAR signaling. (A) Histogram of GSEA Normalized Enrichment Score (NES) highlighting the top 10 differentially expressed Hallmark gene datasets in ΔS and ΔL tumors. (B) Heatmap of type I IFN response gene expression in ΔS and ΔL tumors. (C) Heatmap of gene expression signatures for distinct immune subpopulations in ΔS and ΔL tumors. (D) Relative mRNA expression of representative type I IFN genes (*Ifnb1*, *Ifne*) or type I IFN targets (*Oasl1*, *Isg20*), measured by RT-qPCR. Each dot represents an independent biological replicate (ΔS n = 6, ΔL n = 10). Differences were assessed by an unpaired two-tailed t-test. (E) Experimental design for scRNA-seq analysis of CD45 + cells. CD45 + cells were sorted from three independent ΔS and ΔL tumors, uniquely labeled by antibody-coupled barcoding, pooled, and processed for scRNA-seq analysis. (F) Number of high-quality CD45 + cells recovered from ΔS and ΔL tumors. (G) UMAP of library size per cell. (H) Heatmap of genes used to identify specific subpopulations within CD45 + cells. (I) Distribution of CD45 + cells across different subpopulations in ΔS and ΔL tumors. (J) Average expression of the type I IFN response signature across antigen-presenting populations (B cells, dendritic cells, and macrophages) and CD8 + T cells. Source data

To dissect the mechanisms by which 4C4 deletions influence PDAC phenotypes, we performed RNA-seq on bulk ΔL and ΔS tumors and assessed signaling pathways and immune cell composition using CIBERSORT^41^. Relative to ΔS tumors, ΔL tumors displayed a decrease in pathways linked to IFN signaling (Extended Data Fig. 5a,b), as well as a broad depletion in immune signatures, including B- and T-cell populations (Extended Data Fig. 5c). RT–qPCR confirmed that ΔL tumors have reduced mRNA levels of type I IFNs (*Ifnb1* and *Ifne*) and IFN-responsive genes (*Oasl1* and *Isg20*) (Extended Data Fig. 5d). Single-cell RNA sequencing (scRNA-seq) of tumor-infiltrating CD45^+^ cells isolated from ΔS and ΔL tumors identified changes in the abundance of multiple immune cell populations (Fig. 6a,b and Extended Data Fig. 5e–i). ΔL tumors had fewer B cells and myeloid populations, which was accompanied by increased CD8^+^ T cells (Extended Data Fig. 5i).

**Extended Data Fig. 6:**
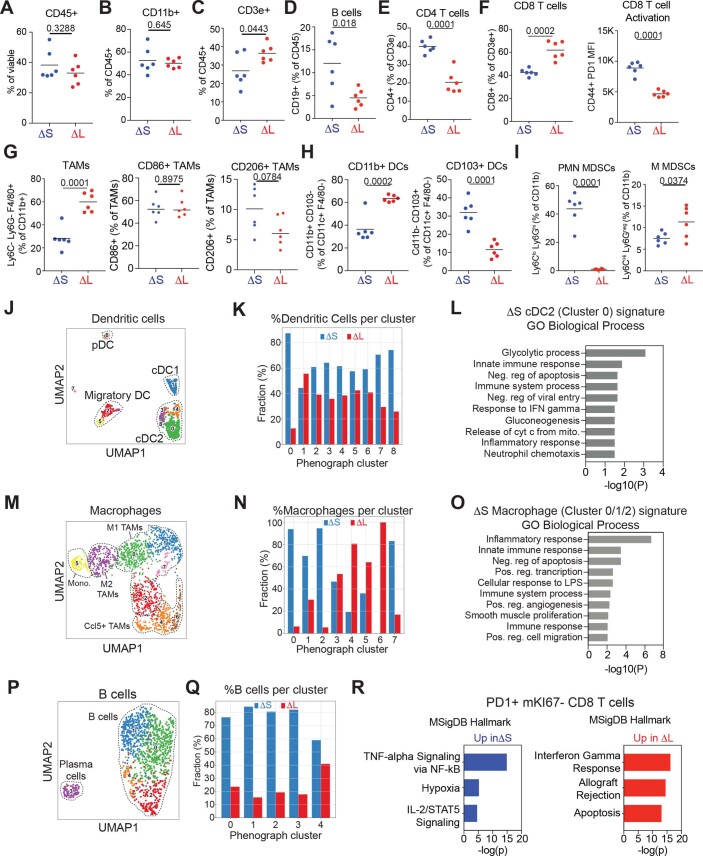
4C4 deletions alter the state of antigen presenting cells. (A-I) Immunophenotyping of infiltrating populations in ΔS and ΔL tumors. Frequency of CD45^+^ cells (A), CD11b^+^ cells (B), CD3e^+^ cells (C), CD19^+^ B cells (D), CD4^+^ T cells (E), CD8^+^ T cells and corresponding PD1 mean fluorescence intensity of CD44^+^CD8^+^ T cells (F), tumor-associated macrophages (TAMs) including CD86 + and CD206 + subtypes (G), CD11b^+^ and CD103^+^ dendritic cell subsets (H), and myeloid-derived suppressor cells (MDSCs) including polymorphonuclear (PMN-MDSCs) and mononuclear (M-MDSCs) subtypes (I). Differences were assessed by unpaired two-tailed t-test. Each dot represents an independent biological replicate (ΔS n = 6, ΔL n = 6). (J) UMAP of dendritic cell phenographs from ΔS or ΔL tumors. Known populations/states are circled. (K) Frequency of dendritic cells across phenographs in ΔS or ΔL tumors. (L) DAVID analysis of Gene Ontology Biological Processes enriched in ΔS-specific dendritic cells in Cluster 0 (green cluster in panel J). (M) UMAP of macrophage phenographs from ΔS or ΔL tumors. Known populations/states are circled. (N) Frequency of macrophages across phenographs in ΔS or ΔL tumors. (O) DAVID analysis of Gene Ontology Biological Processes enriched in ΔS-specific macrophages in clusters 0-2 (green, blue, and red clusters in panel M). (P) UMAP of B cell phenographs from ΔS or ΔL tumors. Known populations/states are circled. (Q) Frequency of B cells across phenographs in ΔS or ΔL tumors. (R) Enrichr analysis of the top Hallmark Pathways enriched in exhausted CD8 + T cells from ΔS and ΔL tumors. Source data

Beyond alterations in the composition of infiltrating CD45^+^ cells, 4C4 deletions led to changes in the transcriptional state of immune subsets. Analysis of an experimentally derived type I IFN response signature (Methods and Supplementary Table 1) showed that professional antigen-presenting cells (APCs; macrophages, dendritic cells and B cells) and CD8^+^ T cells exhibited reduced type I IFN signaling in ΔL tumors (Fig. 6c and Extended Data Fig. 5j). The changes in immune infiltration were confirmed by flow cytometry (Extended Data Fig. 6a–i). The specific effects of 4C4 deletions on APCs were cell type-dependent: a more pro-inflammatory state of cDC2 dendritic cells in ΔS tumors (Extended Data Fig. 6j–l); a shift in macrophage transcriptional states toward higher M1-like cells in ΔS tumors (Extended Data Fig. 6m–o); and an overall reduction across all B-cell subtypes in ΔL tumors (Extended Data Fig. 6p,q).

CD8^+^ T cells showed a range of states, with a dominant presence of activated/exhausted (*Pdcd1*^+^, *Ctla4*^+^, *Havcr2*^+^ and *Lag3*^+^), naive (*Pdcd1*^−^, *Tcf7*^+^ and *Sell*^+^) and cycling cells (*Pdcd1*^+^ and *MKi67*^+^) (Fig. 6d,e). Notably, the non-proliferating *Pdcd1*^+^ population of CD8^+^ T cells occupied distinct phenotypic space in ΔS versus ΔL tumors. Further characterization using Milo^42^ revealed that ΔS tumors accumulated CD8^+^ T cells marked by *Tox* and *Bcl2* expression, whereas those present in ΔL tumors were transcriptionally distinct and displayed higher expression of *Havcr2* and *Lag3* (Fig. 6f–i and Extended Data Fig. 6r). The high levels of IFN-engaged APCs and distinct CD8^+^ T-cell states in ΔS tumors implied ongoing immune surveillance that may suppress metastatic spread. In agreement, depletion of B and CD8^+^ cells, but not CD4^+^ cells, enhanced the metastatic potential of ΔS tumor cells to levels observed for ΔL tumors (Fig. 6j,k). Collectively, these data suggest that loss of tumor-intrinsic type I IFNs impairs the function of APCs and produces a dysfunctional CD8^+^ T-cell state, leading to defects in antitumor immunity.

### 9p21.3 specifies IFN signaling and immune states in human PDAC

**Extended Data Fig. 7:**
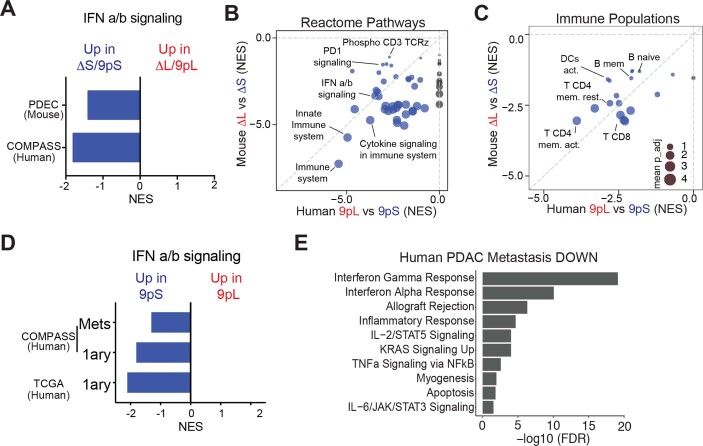
Conserved effects of human 9p21.3 and mouse 4C4 on PDAC immune infiltration. (A) GSEA enrichment scores (NES) of type I IFN signaling in mouse ΔS and human 9pS tumors compared to ΔL and 9pL tumors, respectively. (B) Comparison of GSEA NES scores for Reactome Pathways enriched in mouse ΔS (y axis) and human 9pS tumors (x axis). Highlighted are key pathways and immune populations enriched in IFN-proficient tumors. Circle size represents the adjusted p value (the scale is shown in panel C). Patient data is from primary tumors (9pS n = 37 patients and 9pL n = 28 patients). (C) Comparison of GSEA NES scores and Immune populations enriched in mouse ΔS (y axis) and human 9pS tumors (x axis). Highlighted are key immune populations enriched in IFN-proficient tumors. Circle size represents -log(adjusted p value). Patient data is from primary tumors (9pS n = 37 patients and 9pL n = 28 patients). (D) GSEA enrichment scores (NES) of type I IFN signaling in human primary (1ary) or metastatic (Mets) 9pS tumors compared to 9pL tumors from the COMPASS and TCGA datasets. (E) Hallmark pathways downregulated in human PDAC liver metastases vs. primary tumors. Data from Moffitt et al., 2015^72^.

To evaluate how distinct 9p21.3 deletions alter the tumor microenvironment in human PDAC, we analyzed sequencing data obtained from the COMPASS trial, which contains 218 primary and 180 metastatic PDAC samples isolated by laser capture microdissection^43,44^. Whole-genome and RNA-seq data from these samples allowed tumors to be categorized by 9p deletion status and analyzed for signatures linked to infiltrating immune cells. Consistent with our findings in mice, 9pL deletions in the primary human tumors correlated with reduced type I IFN signaling compared to 9pS alleles (Extended Data Fig. 7a).

The genotype-specific differences in pathways and inferred immune cell composition correlated well across species (Extended Data Fig. 7b,c and Supplementary Table 2). Notably, IFN cluster-proficient (ΔS/9pS) tumors were enriched in pathways associated with innate and adaptive immune infiltration (Extended Data Fig. 7b) and showed a relative enrichment of most immune cell populations, particularly effector CD8^+^ T and B-cell subsets (Extended Data Fig. 7c). Type I IFN signatures present in primary 9pS tumors were, however, reduced in 9pS metastases (Extended Data Fig. 7d)^45^ and analysis of RNA-seq data from a second cohort of matched primary and metastatic PDAC samples confirmed a reduction in type I IFN signaling in metastases irrespective of genotype (Extended Data Fig. 7e). When considered in the context of our functional studies, these data imply that downregulation of type I IFN signaling, by genetic or other means, promotes PDAC metastasis.

### IFNAR1 blockade rescues immune evasion and metastasis

**Fig. 7:**
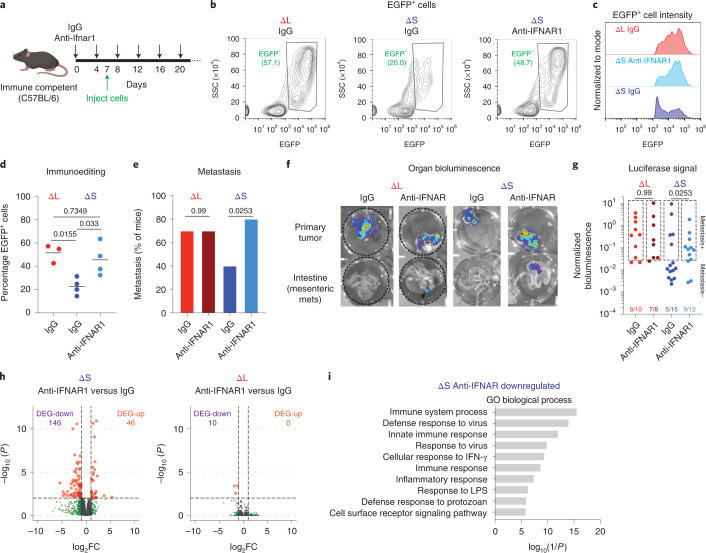
IFNAR1 blockade promotes immune evasion and metastasis. **a**, Experimental outline to test the role of IFNAR1 signaling in transplantation experiments. **b**, Representative flow cytometry plots of ΔS or ΔL tumors from C57BL/6 mice treated with IgG or αIFNAR1 antibodies. SSC, side scatter. **c**, Representative flow cytometry plots of EGFP^+^ populations from IgG ΔL, IgG ΔS or anti-IFNAR1 ΔS tumors. **d**, Quantification of EGFP^+^ cells in ΔS or ΔL tumors from C57BL/6 mice treated with IgG or anti-IFNAR1 antibodies. Each dot represents an independent biological replicate (IgG ΔS *n* = 4, anti-IFNAR1 ΔS *n* = 4, IgG ΔL *n* = 3). Differences were assessed with one-way ANOVA followed by Tukey’s multiple comparison. **e**, Incidence of metastasis in C57BL/6 mice transplanted with homozygous ΔS or ΔL lines and treated with IgG or anti-IFNAR1 antibodies. Two independently generated input cell lines were used per genotype and independent mice were assessed (IgG ΔS *n* = 15, anti-IFNAR1 ΔS *n* = 10, IgG ΔL *n* = 10, anti-IFNAR1 ΔL *n* = 8). Differences were assessed with chi-squared test. **f**, Representative bioluminescent images of primary tumors and intestines from mice with the indicated genotypes of transplanted cells and antibody treatments. **g**, Quantification of all replicates. Boxes indicate signals above the threshold for metastasis detection. Two independently generated input cell lines were used per genotype and independent mice were assessed (IgG ΔS n = 15, anti-IFNAR1 ΔS *n* = 12, IgG ΔL *n* = 10, anti-IFNAR1 ΔL *n* = 8). Differences were assessed with chi-squared test. **h**, Volcano plots of differentially expressed genes (DEGs) comparing IFNAR1 blockade versus IgG controls in ΔS or ΔL tumors. Up, upregulated; down, downregulated. **i**, DAVID Gene Ontology analysis of anti-IFNAR1 downregulated genes in ΔS tumors. Top ten significant pathways are shown. Source data

The immune-evasive and pro-metastatic features of ΔL tumors could plausibly involve other genes beside type I IFNs, notably *Mtap*, whose disruption can influence tumor behavior^46^. To test whether loss of type I IFN signaling is sufficient to cause immune evasion and metastasis as in ΔL tumors, we used blocking antibodies to the type I IFN receptor subunit (IFNAR1) to disrupt type I IFN signaling in the host. Immune-competent mice were pre-treated with an IFNAR1-blocking antibody or an isotype control, followed by orthotopic transplantation of ΔS and ΔL cells, then we analyzed immunoediting of the EGFP-luciferase reporter and overall incidence of metastasis (Fig. 7a).

**Extended Data Fig. 8:**
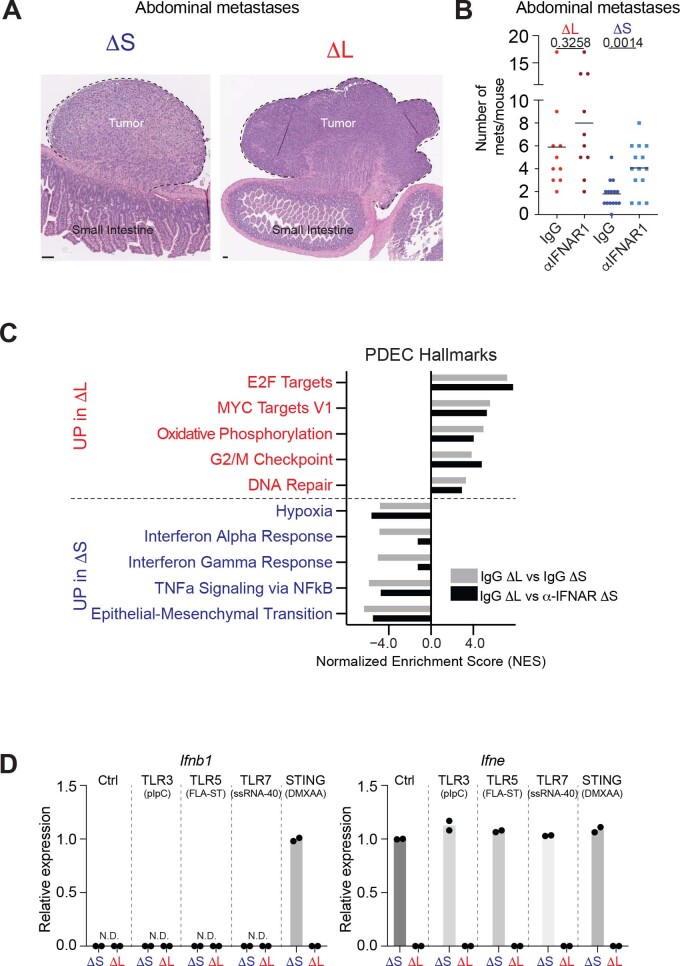
IFNAR1 blockade rescues immune editing and metastasis. (A) Representative H/E images of mesenteric lymph node metastases. Scale bar is 200 μm. (B) Quantification of mesenteric lymph node metastases in mice with the indicated genotypes of transplanted cells and antibody treatments. Differences were assessed by unpaired two-tailed t-test comparing IgG vs αIFNAR1 in the corresponding cell lines. Each dot represents an independent biological replicate (IgG ΔS n = 15, αIFNAR1 ΔS n = 14, IgG ΔL n = 10, αIFNAR1 ΔS n = 10). (C) IFNAR1 blockade specifically affects IFN signaling. NES scores of top 5 UP and DOWN Hallmark categories in tumors comparing ΔL vs ΔS (grey bars, data from Fig. 4C) and NES scores for the same Hallmark categories comparing ΔL vs α−IFNAR1 ΔS (black bars). (D) qRT-PCR measurements of mRNA levels for *Ifnb1* and *Ifne* in ΔS and ΔL tumor-derived cells after the indicated treatments. Dots represent independent cell lines (n = 2). Source data

Consistent with our model, ΔS tumors arising in mice subjected to IFNAR1 blockade expressed higher levels of EGFP than isotype-treated controls (Fig. 7b–d) and showed a greater incidence of metastasis in secondary transplantation assays (Fig. 7e–g and Extended Data Fig. 8a,b). Remarkably, these patterns were comparable to those arising in immune-competent mice receiving ΔL cells and in immune-deficient animals transplanted with ΔS cells (Fig. 4c,d). In contrast, type I IFN blockade had no impact on the already enhanced metastatic potential of ΔL cells (Fig. 7e–g). Transcriptional profiling of bulk tumors confirmed that IFNAR1 blockade phenocopied the reduction of type I IFN signaling observed in IFN-deficient tumors but had minimal impact on the transcriptome of ΔL tumors (Fig. 7h,i and Extended Data Fig. 8c). These data imply that loss of one or more type I IFNs is sufficient to produce the immune-evasive and pro-metastatic phenotypes arising in tumors with homozygous ΔL deletions.

### *Ifne* is a tumor-specific mediator of immunity and metastasis

**Fig. 8:**
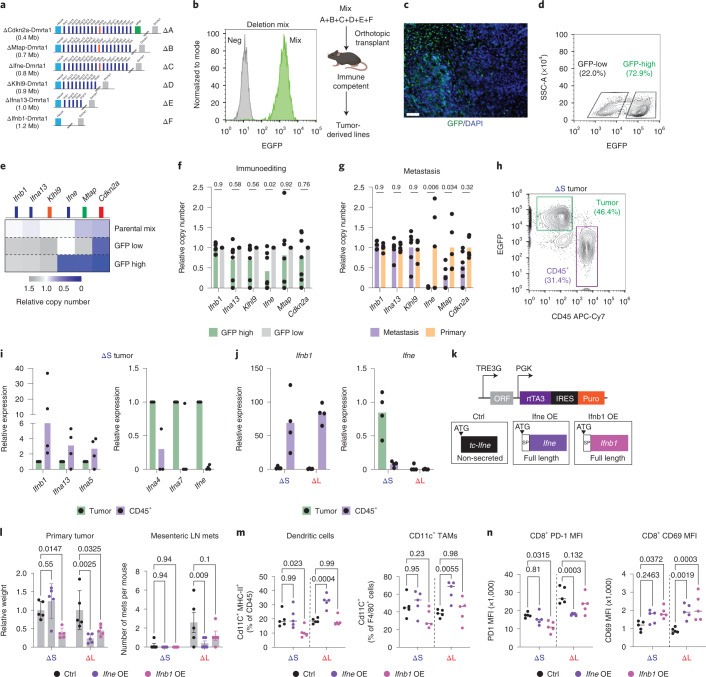
*Ifne* promotes immune surveillance and inhibits metastasis. **a**, Schematic of extended series of 4C4 deletion alleles. **b**, Flow cytometry measurement of EGFP fluorescence in cultured deletion series mix (‘Mix’) (left). EGFP-negative cells were used as negative controls (‘Neg’). Schematic of in vivo competition experiment (right). **c**, Representative EGFP immunofluorescent stain of a deletion-mix tumor, which was repeated in all tumors arising from the in vivo competition assay (*n* = 7). Scale bar, 200 μm. **d**, Representative flow cytometry plot of EGFP levels in a deletion-mix tumor. GFP-low and GFP-high cell populations were sorted as marked. **e**, Copy-number qPCR analysis of the indicated genes in the parental cell mix and GFP-low and GFP-high cells sorted from the tumor from **d**. This was repeated in all tumors arising from the in vivo competition assay (*n* = 7). **f**, Relative copy-number quantification of indicated genes in GFP-high versus GFP-low cells. Differences were assessed by one-way ANOVA followed by Sidak’s multiple comparison test. Each dot is an independent biological replicate (*n* = 7). **g**, Relative copy-number quantification of indicated genes in metastases- versus primary tumor-derived cells. Differences were assessed by one-way ANOVA followed by Sidak’s multiple comparison test. Each dot is an independent biological replicate (primary *n* = 5 and metastasis *n* = 6). **h**, Representative flow cytometry plot of tumor (EGFP^+^) and immune (CD45^+^) cells from a ΔS tumor. **i**, RT–qPCR of the indicated IFN genes in tumor cells and infiltrating CD45^+^ cells from ΔS tumors. Each dot is an independent biological replicate (*n* = 4 tumors). **j**, RT–qPCR of *Ifnb1* and *Ifne* in tumor cells and infiltrating CD45^+^ cells from ΔS and ΔL tumors. Each dot is an independent biological replicate (ΔS *n* = 4 and ΔL *n* = 4). **k**, Design of the vector for doxycycline-inducible expression of full-length mouse *Ifnb1*, *Ifne* or a truncated version. **l**, Relative quantification of primary tumor weights (left) and number of mesenteric LN metastases (right). Differences were assessed by one-way ANOVA followed by Sidak’s multiple comparison test to the respective control population. Each dot is an independent biological replicate (*n* = 5 for all conditions). **m**, Frequency of dendritic cells (left) and Cd11c^+^ TAMs (right). Differences were assessed by one-way ANOVA followed by Sidak’s multiple comparison test to the respective control population. Each dot is an independent biological replicate (*n* = 5 for all conditions). **n**, Levels of PD-1 (left) and CD69 (right) levels in CD8^+^ T cells. Differences were assessed by one-way ANOVA followed by Sidak’s multiple comparison test to the respective control population. Each dot is an independent biological replicate (*n* = 5 for all conditions). Source data

The functional redundancy between different type I IFNs remains poorly understood^47^. For instance, *Ifnb1* is highly expressed in immune cells and is a key effector of the cGAS-STING pathway in engaging innate and adaptive immunity, but the relative contributions of most other type I IFNs to immunity are unclear^26,48^. To dissect the functional contribution of different tumor-derived IFNs to immunoediting and metastasis, we leveraged MACHETE to engineer a refined deletion series that encompass a gradually increasing number of IFN genes (Fig. 8a), resembling deletions seen in cancer patients. The resulting cell populations were orthotopically injected as pools into immunocompetent recipient mice (Fig. 8b) and expression of EGFP-luciferase reporter was used as an indicator of immune evasion in the resulting tumors.

Consistent with variable degrees of immune evasion, tumors showed heterogenous expression of EGFP (Fig. 8c). Cells with low versus high levels of EGFP were isolated and the EGFP-high population showed enrichment of deletions affecting the IFN cluster (Fig. 8d), with a significant enrichment of cells harboring deletions of *Ifne* across multiple independent tumors (Fig. 8e,f). A similar increase in the deletion of *Ifne* was observed when comparing metastases to primary tumors (Fig. 8g), further highlighting the potential relevance of *Ifne* to tumor dissemination.

A detailed analysis of type I IFN gene expression in epithelial and CD45^+^ immune cells present in ΔS tumors reinforced the above observations. As previously reported, *Ifnb1* could be induced in vitro by a cGAS-STING agonist, yet in vivo it was more highly expressed in CD45^+^ cells than in tumor cells; by contrast, other IFNs, particularly *Ifne*, were not induced by these stimuli in vitro and showed preferential expression in tumor cells (Fig. 8h–j and Extended Data Fig. 8d). Collectively, these data imply that disruption of the tumor-specific *Ifne* is necessary for the effects of type I IFN cluster loss on immune evasion and metastasis.

**Extended Data Fig. 9:**
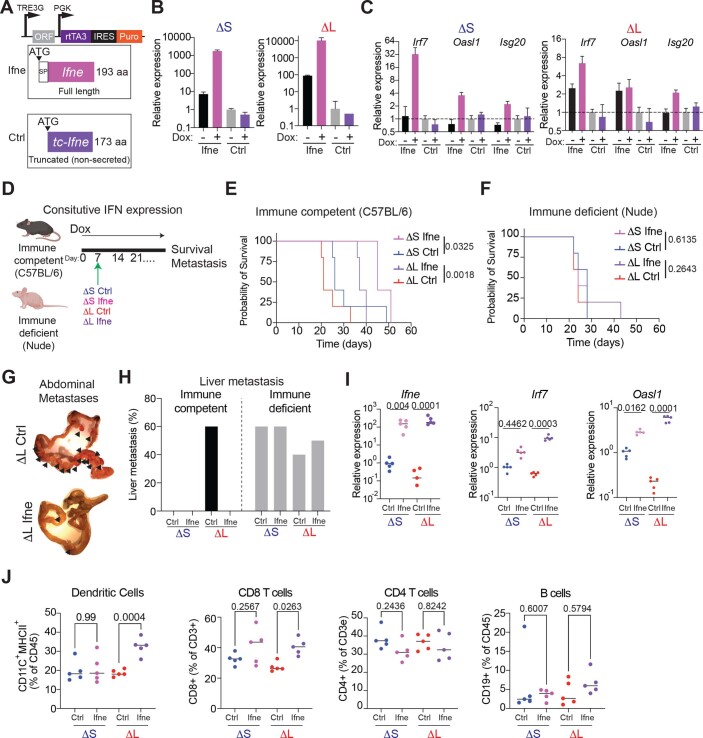
Ifne overexpression blunts metastasis by engaging adaptive immunity. (A) Design of the vector for doxycycline-inducible expression of full-length mouse *Ifne* or, as control, a truncated version lacking the signal peptide. (B) Representative RT-qPCR of *Ifne* expression in cells cultured -/+ doxycycline (2 μg/mL) for 72 hours. The assay specifically amplifies full-length *Ifne*. Bars represent median and upper/lower limits. Experiment was repeated n = 2 with similar results. (C) Representative RT-qPCR of IFN target genes (*Irf7*, *Oasl1*, *Isg20*) in cells cultured -/+ doxycycline (2 μg/mL) for 72 hours. Bars represent median and upper/lower limits. Experiment was repeated n = 2 with similar results. (D) Experimental design to test the role of sustained *Ifne* expression in immune competent and immune deficient mice. (E) Survival curve of immune competent mice orthotopically transplanted with Ctrl or *Ifne* overexpressing ΔS and ΔL cells. n = 5 independent biological replicates. Differences were assessed by log rank test. (F) Survival curve of immune deficient (nude) mice orthotopically transplanted with Ctrl or *Ifne* overexpressing ΔS and ΔL cells. n = 5 independent biological replicates. Differences were assessed by log rank test. (G) Representative image of an intestine from a mouse with sustained expression of Ctrl or full-length *Ifne* ΔL cells at endpoint. Arrowheads point to macrometastases in the mesentery and intestine. (H) Incidence of overt liver metastasis in immune proficient and immune deficient hosts transplanted with ΔS or ΔL cells expressing Ctrl or full-length *Ifne* (n = 5 independent biological replicates). (I) RT-qPCR of *Ifne*, *Irf7*, and *Oasl1* in tumors from immune competent mice treated with doxycycline for 1 week before tumor analysis. Each dot represents a tumor from an independent mouse (n = 5 independent biological replicates). Differences were assessed by one-way ANOVA followed by Sidak’s multiple comparison test. (J) Frequency of dendritic cells (far left), CD8 T cells (left), CD4 T cells (right), and B cells (far right) are shown. Each dot represents a tumor from an independent mouse (n = 5 independent biological replicates). Differences were assessed by one-way ANOVA followed by Sidak’s multiple comparison test. Source data

**Extended Data Fig. 10:**
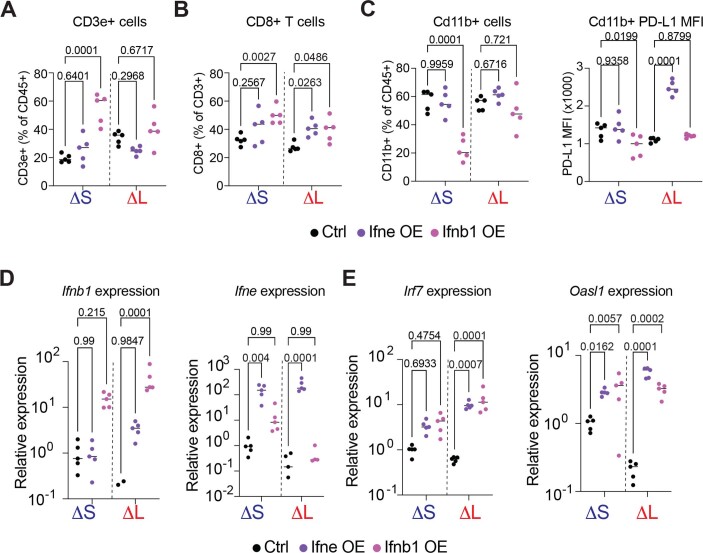
Ifne and Ifnb1 show distinct effects on immune infiltrating cells. (A) Frequency of T cells (CD3e + ). Differences were assessed by one-way ANOVA followed by Sidak’s multiple comparison to the respective control population. Each dot is a tumor from an independent mouse (n = 5 independent biological replicates). (B) Frequency of CD8 + T cells from ΔS and ΔL tumors treated for one week with Dox. Differences were assessed by one-way ANOVA followed by Sidak’s multiple comparison to the respective control population. Each dot is a tumor from an independent mouse (n = 5 independent biological replicates). Control and *Ifne* data are also used in Extended Data Fig. 9J. (C) (Left) Frequency of CD11B + cells (CD11B + CD3e-); (Right) Median PD-L1 surface expression in CD11B + cells, from ΔS and ΔL tumors treated for one week with Dox. Differences were assessed by one-way ANOVA followed by Sidak’s multiple comparison to the respective control population. Each dot is a tumor from an independent mouse (n = 5 independent biological replicates). Control and *Ifne* data are used in Extended Data Fig. 9J. (D-E) Relative expression of *Ifnb1*, *Ifne* (D); *Irf7*, and *Oasl1* (E) in ΔS and ΔL tumors with add-back of *Ifnb1*, *Ifne*, or control construct. Differences were assessed by one-way ANOVA followed by Sidak’s multiple comparison to the respective control population. Each dot is a tumor from an independent mouse (n = 5 independent biological replicates). Control and *Ifne* data are also used in Extended Data Fig. 9. Source data

To determine whether *Ifne* is sufficient to suppress immune evasion and metastasis, we introduced a doxycycline-inducible construct to express either full-length or a truncated *Ifne* in ΔS and ΔL cells (Extended Data Fig. 9a–c). Sustained induction of full-length *Ifne* suppressed overt metastasis of ΔL tumors, which was dependent on adaptive immunity (Extended Data Fig. 9d–h). Both ΔS and ΔL showed the expected overexpression of *Ifne* and downstream type I IFN target genes (Extended Data Fig. 9i); nevertheless, these two genotypes showed differential response to acute *Ifne*: ΔS tumors had no response while ΔL tumors had a reduction in tumor size and metastasis (Fig. 8k,l). Consistent with a loss-of-function phenotype in ΔL tumors, enforced *Ifne* expression in these tumors resulted in elevated levels of APCs and activated CD8^+^ T cells (Fig. 8m,n and Extended Data Fig. 9j). The *Ifne* effects were distinct from those produced by *Ifnb1*, which inhibited primary tumor growth in both ΔS and ΔL tumors but was unable to efficiently suppress the metastasis of ΔL tumors (Fig. 8l). In contrast to *Ifne*, *Ifnb1* did not elicit a reduction on CD8^+^ T-cell PD-1 levels and did not affect APCs (Fig. 8m,n and Extended Data Fig. 10a–c), despite activating downstream targets to a similar extent (Extended Data Fig. 10d,e). These data suggest that different type I IFNs have non-redundant impact on the tumor microenvironment, whereby the STING-independent *Ifne* can remarkably play a dominant role.

Taken together, our results demonstrate that somatic deletion of type I IFNs impairs immunoediting and derepresses metastasis and reveal a previously unappreciated role of *Ifne* in suppressing tumor immune evasion and metastasis via the adaptive immune system.

## Discussion

Despite the pervasive nature of CNAs across cancers, their functional characterization has been limited by the difficulty of manipulating large genomic regions. MACHETE addresses this challenge: it is a customizable and efficient method, enables engineering deletions of at least 45 Mb, requires no cloning of targeting vectors, eliminates cells with off-target integrations, and allows for engineering allelic series of deletions. Using MACHETE, we reveal unappreciated but clinically relevant insights into the multifactorial nature of 9p21.3 deletions, an event that contributes to up to 15% of human cancers^49^. Given the emerging view that CNAs influence cancer phenotypes by altering the dosage of multiple genes, tools such as MACHETE will be essential for understanding their biology and any therapeutic opportunities they create.

Our results revise the paradigm for how the 9p21.3 locus suppresses tumorigenesis. Most studies have focused on *CDKN2A* (encoding p16^INK4a^ and p14^ARF^) and *CDKN2B* (encoding p15^INK4b^), which potently suppress tumorigenesis by driving cell cycle arrest^17^. Herein, we show that the type I IFN cluster is co-deleted with *CDKN2A/B* in nearly half of all tumors harboring 9p21.3 deletions and, while other 9p21.3 genes such as *Mtap*^46^ may also influence tumor behavior, type I IFNs are critical tumor suppressors. Therefore, 9p21.3 deletions not only disable a potent block to cell proliferation but also facilitate immune evasion, simultaneously disrupting cell-intrinsic and cell-extrinsic tumor-suppressive programs.

Our findings indicate that *Cdkn2a* loss is a requisite event that enhances proliferative capacity while co-deletion of type I IFNs provides a collateral benefit that promotes immune evasion. This model also explains why type I IFN expression from neighboring cells is unable to compensate for these deletions, as incipient tumors may eventually reach a size where paracrine IFN signaling becomes ineffective. Regardless, the ability of tumor cells harboring type I IFN deletions to avoid immune surveillance at the primary tumor site increases their metastatic potential. As such, the type I IFN cluster acts as a bona fide metastasis suppressor locus, adding support to the emerging view that immune surveillance plays an important role in limiting metastatic spread.

The role of different tumor-derived type I IFNs during cancer progression has remained unclear, with most attention given to IFN secretion by immune cells or the regulation of *Ifna/b* genes downstream of cGAS-STING signaling^48,50–52^. Nonetheless, in our immune-evasive ΔS model, a subset of type I IFNs, particularly *Ifne*, are exclusively expressed in tumor cells, where they promote type I IFN signaling and dictate the composition and state of immune cell infiltrates. Consequently, deletion of the type I IFN cluster produces a tumor microenvironment that drives with the accumulation of exhausted CD8^+^ T cells that express markers of terminal differentiation, analogous to those observed in *Ifnar1* knockout mice during defective responses to pathogen challenge^53^. The lack of responsiveness of *Ifne* to classic type I IFN inducers (such as TLR and cGAS-STING agonists) highlights its potential function as a constitutive enforcer of tumor immune surveillance, perhaps mirroring its only known role in mediating mucosal immunity^54^. Our data also uncover unique effects of distinct IFNs on infiltrating immune cells, which are modified by the genetic status of the 9p21 locus and argue for complex interplay of these molecules to engage the IFN pathway across cell types.

Beyond fundamental insights, our study has clinical implications for the stratification of patients receiving ICB therapy, which has well-known heterogeneous patterns of response. To date, successful responses have been associated with tumor mutational burden^55^, patterns of immune infiltration^56^ and specific genetic alterations^22,23,57^. Among the genetic alterations, 9p21.3 deletions have been recurrently associated with lack of response, but the mechanistic basis for these observations has remained unclear^22,23,57^. Our data implicate the type I IFN cluster as a key determinant of ICB response within the locus, yet current clinical platforms for targeted sequencing^58^ probe only *CDKN2A/B* and not the IFN cluster. We envision that the incorporation of type I IFN status will aid patient stratification in melanoma and potentially other cancers with recurrent 9p21.3 deletions.

In summary, our results nominate type I IFN deletions as a pervasive genetic mechanism of immune evasion in cancer, rivaling the deletions of the human leukocyte antigen (HLA) cluster^59^. Whether the linkage between IFNs and *Cdkn2a/b* is biologically meaningful remains to be determined, but it is noteworthy that both type I IFNs and *Cdkn2a*-encoded proteins limit viral infection^47,60^, which may have been co-opted for tumor suppression. Of note, genome-wide association studies have identified the 9p21.3 locus as one of two highly significant regions that are broadly associated with multiple age-related pathologies, the other key region coinciding with the HLA locus on chromosome 6p21 (ref. ^24^). While *CDKN2A* is thought to drive the 9p associations, our study raises the possibility that variation in type I IFN regulation plays a role in the biology of these pathologies as well.

## Methods

### Statistics and reproducibility

Graphs and statistical analyses for Figs. 3–8 and Extended Data Figs. 3–6 and 8–10 were conducted with GraphPad Prism. For all experiments n represents the number of independent biological replicates. For Figs. 3d and 4g and Extended Data Figs. 3i, 4d, 5d and 6a–i, differences were evaluated with an unpaired two-tailed Student’s *t*-test. For Figs. 5d,e,h, 7d,g and 8f,g,l–n and Extended Data Figs. 4c–i, 8b, 9i,j and 10a–e, differences were assessed by a one-way ANOVA followed by Tukey or Sidak’s multiple comparison test compared to each genotype’s control condition. For tumor growth kinetics in Fig. 5g, a two-way ANOVA followed by Sidak’s multiple comparison test was used. To assess differences in tumor initiation or metastasis incidence, contingency tables followed by Fisher’s exact test or chi-squared test were conducted for Figs. 3a, 4c–e, 4g, 4j, 4m, 6k and 7e,g and Extended Data Fig. 3h,k. For survival curves, a log-rank test was used to assess significant differences for Figs. 3f and 4a,h and Extended Data Fig. 9e,f. Randomization was used to allocate mice to treatments with ICB (Fig. 5) when the primary tumor reached a 5 mm diameter (PDEC model) or 100 mm^3^ (B16F10 model). Metastasis quantification was performed blinded and no data were excluded. For experiments showing representative data, experiments were repeated at least three times and showed consistent results.

### Pan-cancer TCGA data analysis

Analysis of TCGA datasets was performed on cBioPortal^61^. All TCGA datasets were selected and the onco-query language entry shown in Supplementary Table 3 was used to identify tumors with 9p21.3 deletions. Tumors with at least 10% of patients harboring 9p21.3 deletion were identified. Tumors were classified as 9pS if they had a focal deep deletion of *CDKN2A/B*. Tumors were classified as 9pL if both *CDKN2A/B* and the type I IFN cluster was deleted. For the 9pL/9pS relative frequency, only datasets with at least 40 cases with 9p21.3 loss were considered.

### Cell culture

NIH3T3 fibroblasts were obtained from the American Type Culture Collection (ATCC) and were cultured in DMEM supplemented with 10% fetal bovine serum (FBS) and 100 IU ml^−1^ of penicillin/streptomycin. Parental and stably expressing Gag/Pol HEK293 lines were cultured in DMEM supplemented with 10% FBS and 100 IU ml^−1^ of penicillin/streptomycin. PDECs, derived from female C57BL/6n mice, were cultured as previously described^33,34^ in Advanced DMEM/F12 supplemented with 10% FBS (Gibco), 100 IU ml^−1^ of penicillin/streptomycin (Gibco), 100 mM Glutamax (Gibco), ITS supplement (Sigma), 0.1 mg ml^−1^ soy trypsin inhibitor (Gibco), bovine pituitary extract (Gibco), 5 nM triiodothyronine (Sigma), 100 μg ml^−1^ cholera toxin (Sigma), 4 μg ml^−1^ dexamethasone (Sigma) and 10 ng ml^−1^ human EGF (Preprotech). PDECs were cultured on collagen-coated plates (100 μg ml^−1^ PureCol 5005, Advanced Biomatrix).

Tumor-derived cell lines were generated by an initial mechanical disaggregation/mincing and tumor fragments were transferred to a solution of type V collagenase (Sigma C9263, 1 mg ml^−1^ in HBSS 1×) and incubated at 37 °C for 45 min. Cell suspensions were supplemented with an equal volume of DMEM 10% FBS and filtered through a 100-μm mesh (BD). Filtered suspensions were centrifuged for 5 min at 300*g*, pellets were resuspended in DMEM 10% FBS with penicillin/streptomycin 100 μ ml^−1^ and cultured on collagen-coated plates (100 μg ml^−1^ PureCol 5005, Advanced Biomatrix). Cells were passaged twice to remove non-tumor cells and the resulting tumor-derived cells were used for subsequent applications.

B16F10 cells were obtained from ATCC and cultured in DMEM supplemented with 10% FBS and 100 IU ml^−1^ of penicillin/streptomycin.

### Engineering large genomic deletions with MACHETE

To engineer genomic deletions, we developed MACHETE. The premise behind MACHETE is to give cells that bear the deletion of interest a selective advantage over unedited cells, which is achieved by using a bicistronic cassette consisting of an inducible suicide element and an antibiotic resistance component. This cassette is integrated into the region of interest by CRISPR-Cas9 mediated homology-directed repair (HDR). Cells with stable integration of the cassette are positively selected, then treated with CRISPR-Cas9 to generate the deletion of interest, and edited cells are enriched via negative selection.

#### Identification and in vitro transcription of sgRNAs

We used GuideScan to select optimal sgRNA sequences^62^. For each locus of interest, we identified an sgRNA to introduce the MACHETE cassette by HDR and sgRNAs to generate the deletion of interest. For the 4C4 locus, we designed two independent sets of guides for each deletion to control for potential off-target effects. We generated sgRNAs as previously described^63^. Briefly, a primer with a T7 adaptor and the sgRNA sequence was used to PCR amplify the tracrRNA from a pX330 plasmid. The PCR product was then purified and transcribed using the RNA MAXX In Vitro Transcription kit (Agilent) to produce the sgRNA. sgRNAs were then column purified (RNA Clean & Concentrator, Zymo Research), eluted in water and aliquoted for later use with recombinant Cas9 (Sigma). Oligonucleotides used for sgRNA production are listed in Supplementary Table 4.

#### Generation of HDR donor

To maximize flexibility, MACHETE uses 40-bp homology arms that are introduced by PCR. The locus-specific HDR donors were generated by PCR amplification of the MACHETE bicistronic cassette using a high-fidelity DNA polymerase (Herculase II, Agilent or Q5, NEB). PCR fragments were column purified (QIAGEN) and quantified. Primers for targeting are presented in Supplementary Table 4.

#### CRISPR-Cas9-mediated targeting and generation of large genomic deletions

For all CRISPR editing, we used Cas9 RNPs with the intended guides, to reduce cloning and limit Cas9 expression. To incorporate Cas9 RNPs and donor PCR product, cells were electroporated with the Neon System (Invitrogen) following the manufacturer’s instructions.

#### HDR knock-in of MACHETE cassette

Briefly, cells were trypsinized, washed once in PBS and counted. Cells were then resuspended in Neon Buffer R and aliquoted for the different electroporation reactions. Each condition used 100 × 10^3^ cells in 10 μl of Buffer R. In parallel, 1 μg of Cas9 (Thermo Fisher) and 1 μg of sgRNA were complexed for 15 min at room temperature. For the HDR step, 0.5 μg of donor DNA was added to the Cas9 RNP complex, which was then mixed with the cell aliquot. The cell–RNP–donor mixture was electroporated (1,400 V pulse voltage, 20 ms pulse width, two pulses). For the selection of cassette KI lines, puromycin (2 μg ml^−1^) was added to the medium 48 h after electroporation. In the case of fluorescence reporters, cells were sorted 48 h after electroporation (Sony MA900) and further enriched for stable expression 1 week after this initial sort. Selected cells were expanded to establish the parental KI lines. To validate this initial step, cells were then treated with DT (50 ng ml^−1^) or ganciclovir (10 μg ml^−1^) to assess their sensitivity. On-target integrations were assessed by PCR of gDNA and Sanger sequencing of the product for confirmation. Genotyping primers are provided in Supplementary Table 4.

#### Generation of genomic deletions

KI cells were trypsinized, washed in PBS once and counted. Cells were then resuspended in Neon Buffer R and aliquoted for the different electroporation reactions. Each condition used 10^5^ cells in 10 μl of Buffer R. In parallel, 2 μg of Cas9, 1 μg of 5′ flanking sgRNA and 1 μg of 3′ flanking sgRNA were complexed for 15 min at room temperature. The cell/RNP mixture was electroporated (1,400 V pulse voltage, 20 ms pulse width, two pulses) and cells were seeded in the absence of selection. At 48 h after seeding, cells were treated with DT (50 ng ml^−1^) or ganciclovir (10 μg ml^−1^) and medium was changed every 2 d with ongoing selection. Surviving cells were then passaged and analyzed for the presence of the intended deletion breakpoint and loss of selection cassette and sensitivity to selection was re-evaluated. Genotyping primers are provided in Supplementary Table 4.

#### Breakpoint high-throughput sequencing

Breakpoint PCR products were purified and sent for amplicon sequencing (Amplicon-EZ, Genewiz) following service guidelines. Raw fastq reads were aligned to the mouse genome (mm10) using bowtie2 with parameters ‘–local -D 50 -R 3 -N 0 -L 19 -i S,1.0,0.7–no-unal -k 5–score-min C,20’. Aligned SAM reads were processed using custom Rscript to parse the breakpoint location, junction position, direction of the reads and alignment types. Alignments for a proper break read-pairs had to meet three criteria: both aligned to the same chromosome; coming from one primary and one secondary alignment; and breakpoints located on opposite sides of the breakpoint junction.

### Flow cytometry

#### Assessing expression of EGFP

Tumor cell suspensions were generated by initial mechanical disaggregation/mincing. Tumor fragments were then transferred to a solution of type V collagenase (Sigma C9263, 1 mg ml^−1^ in 1× HBSS) supplemented with soy trypsin inhibitor (Gibco, 0.1 mg ml^−1^) and DNase I (Sigma, 0.1 mg ml^−1^). Tumor pieces in this disaggregation buffer were transferred to a GentleMACS tube and loaded into the OctoDissociator (Miltenyi). Samples were treated with the mTDK1 program, after which 5 ml of FACS buffer (PBS 1×, 2% FBS) was added to the sample and the mix was filtered through a 100-μm mesh (BD). The resulting cell suspension was centrifuged and resuspended in FACS buffer. Cells were then treated with Fc block (BD, 1:200 dilution) incubated at 4 °C for 15 min and stained with anti-CD45 AF700 (BD, 1:400 dilution) for 30 min at 4 °C. Cells were washed and resuspended in FACS buffer supplemented with DAPI (Sigma, 1 μg ml^−1^ final). Stained cell suspensions were then analyzed in an MA900 sorter (Sony). EGFP^+^ cells were analyzed within the CD45^−^DAPI^−^ population.

#### Multi-parametric flow cytometry analysis

Tumor cell suspensions were generated as above and cells were stained with LIVE/DEAD fixable viability dye (Invitrogen) for 30 min at 4 °C. After this, cells were washed, incubated with Fc block (BD, 1:200 dilution) for 15 min at 4 °C and then stained with conjugated antibody cocktails (the Reporting summary describes antibody panels) for 30 min at 4 °C. After staining, cells were washed and fixed (BD Cytofix for panels with only surface markers; eBioscience Foxp3/Transcription Factor Staining buffer set for panels with intracellular staining) for 20 min at 4 °C. Cell preparations were further stained for intracellular markers when necessary, further washed and stored for analysis. Samples were analyzed in a BD LSR Fortessa with five lasers, where gates were set by use of fluorescence-minus-one controls.

### Animals and in vivo procedures

#### Animals

All mouse experiments were approved by the Memorial Sloan Kettering Cancer Center (MSKCC) Institutional Animal Care and Use Committee. Mice were maintained under pathogen-free conditions and food and water were provided ad libitum. C57Bl/6n and NSG mice were purchased from Envigo. Foxn1^nu^ (Swiss nude) mice were purchased from Jackson Laboratory. All mice used were 6–8-week-old females.

#### PDAC GEMM embryonic stem cell models of Cdkn2a/b loss

Embryonic stem cells (ESCs) bearing alleles to study PDAC were used as previously described^64,65^. Briefly, Ptf1a^Cre/+^; Rosa26^Lox-Stop-Lox rtTA3-IRES-mKate2/+^; Col1a1^Homing cassette/+^ cells were targeted with short hairpin RNAs against *Smad4* or *Renilla* luciferase (non-targeting control). Mice were then generated by blastocyst injection of shSmad4 or shRen ESCs and short hairpin RNAs were induced by treatment of the resulting mice with doxycycline in drinking water starting at 5–6 weeks of age. Pancreatic tumor initiation and progression were monitored by palpation and ultrasound imaging. Mice were killed upon reaching humane endpoints of tumor burden and samples were collected from primary tumors and metastases (when present). Tumor-derived cell lines were then analyzed by sparse whole-genome sequencing and classified according to the type of *Cdkn2a/b* alteration.

#### Orthotopic and subcutaneous transplants

For orthotopic transplants of PDEC cells, mice were anesthetized and a survival surgery was performed to expose the pancreas, where either 300,000 cells (for primary MACHETE-edited lines) or 100,000 cells (tumor-derived lines) were resuspended in a 1:1 PBS/Matrigel mix (Corning) and injected in the pancreas of each mouse. Mice were then monitored for tumor engraftment (bioluminescence imaging, IVIS) and progression. For transplants of B16F10 melanoma cells, mice were anesthetized and 200,000 cells resuspended in a 1:1 PBS/Matrigel mix were injected subcutaneously. For treatment experiments, mice with orthotopic PDAC transplants were monitored for tumor growth by ultrasound. When tumors reached a diameter of ~3–5 mm, mice were randomized and enrolled in the different treatment arms. Mice with melanoma transplants were monitored for tumor growth with a caliper. When tumors reached a volume of ~80–100 mm^3^, mice were randomized and enrolled for the different treatment arms. Maximum tumor burden was established following IRB guidelines: when a tumor reached 10% of weight (PDEC models), reached 1,500 mm^3^ (B16F10 models), or mice had overt disease or signs of distress. All mice reaching any of these end point criteria were killed.

#### Experimental metastasis assays

For liver colonization of PDEC cells, mice were anesthetized and a survival surgery was performed to expose the spleen, where 100,000 cells from tumor-derived lines were injected in the spleen of each mouse. The site of injection was then removed and the remainder of the spleen was cauterized (hemi-splenectomy). Mice were then monitored for tumor engraftment and progression and were killed when overt disease was present in accordance with Institutional Animal Care and Use Committee guidelines.

#### Antibody and drug treatments

For IFNAR1 blockade experiments, mice were treated twice per week with either 200 µg intraperitoneally (i.p.) of control IgG (MOPC21 clone, BioXCell) or 200 µg i.p. of anti-IFNAR1 antibody (MAR15A3, BioXCell). For depletion experiments: mice were treated with anti-CD8a antibody (Clone 2.43, BioXCell) or anti-CD4 (Clone GK1.5, BioXCell) with an initial dose of 400 µg i.p., followed by maintenance injections of 200 µg per mouse. Control, IFNAR1-blocking and CD8/CD4 depletion antibody treatments were conducted twice per week, starting 1 week before the orthotopic transplantation of cells. Treatments were maintained for the entire duration of the experiment. B-cell depletion was performed by a monthly intravenous injection of anti-CD20 (Clone SA271G2, BioLegend), starting 1 week before orthotopic transplantation of cells. For drug response experiments: PDAC-bearing mice were treated with trametinib (1 mg kg^−1^) and palbociclib (100 mg kg^−1^) via oral gavage for four consecutive days followed by 3 d off treatment. Anti PD-1 (200 μg per mouse, clone RMP1, BioXCell) was given three times per week. Melanoma-bearing mice were treated with vehicle of anti-CTLA4 (200 µg per mouse, clone 9H10, BioXCell) three times per week. For IFN-inducible constructs, mice were fed ad libitum with doxycycline-containing chow (200 ppm, Teklad) constitutively (starting 1 week before injection until the end of the experiment) or acutely (starting 2 weeks after injection for 1 week).

#### In vivo bioluminescence imaging

Mice were anesthetized and hair over the imaging site was removed. Mice were injected with 200 µl of luciferin i.p. (200 mg l^−1^, PerkinElmer 122799) and bioluminescence was acquired 10 min after the luciferin injection in an IVIS Spectrum. For organ imaging, mice were injected with luciferin, killed 10 min after the injection and organ bioluminescence was acquired in an IVIS Spectrum instrument.

#### Imaging and assessment of metastatic burden

Mice meeting end point criteria were killed and inspected for overt macro-metastatic burden in the abdominal cavity (peritoneum, diaphragm, mesenteric lymph nodes, ovary/fallopian tubes, kidneys and liver), as well as in the thoracic cavity (lungs and rib cage). Primary tumors and organs were dissected and imaged under a dissection microscope (Nikon SMZ1500) for brightfield and EGFP fluorescence.

### RNA extraction and cDNA preparation

RNA was extracted by using the Trizol Reagent (Thermo Fisher) following the manufacturer´s instructions. The only modification was the addition of glycogen (40 ng ml^−1^, Roche) to the aqueous phase to visualize the RNA pellet after precipitation. RNA was quantified using a Nanodrop. Complementary DNA was prepared with the AffinityScript QPCR cDNA Synthesis kit (Agilent) following the manufacturer’s instructions.

### DNA extraction

Genomic DNA was extracted from cells or tissues using the DNeasy Blood and Tissue kit (QIAGEN) following the manufacturer’s instructions.

### qPCR

For quantitative PCR, the PerfeCTa SYBR Green FastMix (QuantaBio), the Taqman Fast Advanced Master Mix (Applied Biosystems) and the Taqman Genotyping Master Mix (Applied Biosystems) were used following the manufacturer’s instructions. For qPCR primers and Taqman assays, see Supplementary Table 5.

### Histology

Tissues were formalin-fixed, dehydrated and paraffin-embedded for sectioning. Hematoxylin & eosin staining was performed with standard protocols.

### RNA-seq, differential gene expression and gene set enrichment analysis

Bulk tumor pieces were flash frozen on dry ice and stored at −80 °C. Tissues were then mechanically disrupted in Trizol and RNA was extracted following the manufacturer’s instructions. RNA integrity was analyzed with an Agilent 2100 Bioanalyzer. Samples that passed quality control were then used for library preparation and sequencing. Samples were barcoded and run on a HiSeq (Ilumina) in a 76-bp SE run, with an average of 50 million reads per sample. RNA-seq data were then trimmed by removing adaptor sequences and reads were aligned to the mouse genome (GRCm38.91; mm10). Transcript counts were used to generate an expression matrix. Differential gene expression was analyzed by DESeq2 (ref. ^66^) for 3–5 independent tumors per condition. Principal components analysis (PCA) and DEG analysis was performed in R, with significance determined by >twofold change with an adjusted *P* value <0.05. Gene set enrichment analysis (GSEA)^67,68^ was performed using the GSEAPreranked tool for conducting GSEA of data derived from RNA-seq experiments (v.2.07) against specific signatures: Hallmark Pathways, Reactome Pathways and Immune Subpopulations.

### Sparse whole-genome sequencing

Low-pass whole-genome sequencing was performed on gDNA freshly isolated from cultured cells as previously described^69^. Briefly, 1 μg of gDNA was sonicated on an E220 sonicator (Covaris; settings: 17Q, 75s) and libraries were prepared by standard procedure (end repair, addition of polyA and adaptor ligation). Libraries were then purified (AMPure XP magnetic beads, Beckman Coulter), PCR enriched and sequenced (Illumina HiSeq). Reads were mapped to the mouse genome, duplicates removed and an average of 2.5 million reads were used for CNA determination with the Varbin algorithm^70^.

### Human PDAC transcriptional analysis

Samples from the COMPASS trial^43,44^ were classified as primary or metastatic disease and further subdivided by status of the 9p21.3 locus: 9pS deletion affecting *CDKN2A/B* or 9pL deletions affecting *CDKN2A/B* and at least one IFN gene from the linked cluster. 9pS and 9pL samples were then analyzed for DEGs using DESeq2 and assessed by GSEA for Reactome Pathways^71^ and Immune Subpopulations^41^. As an independent validation of the differences between primary and metastatic PDAC, a previously published dataset^72^ was used to derive DEGs using DESeq2. Genes downregulated in PDAC metastasis were then analyzed using the Enrichr algorithm^73^.

### scRNA sequencing

The scRNA-seq of FACS-sorted cell suspensions was performed on a Chromium instrument (10x Genomics) following the user guide manual for 3′ v.3.1. In brief, FACS-sorted cells were washed once with PBS containing 1% bovine serum albumin (BSA) and resuspended in PBS containing 1% BSA to a final concentration of 700–1,300 cells per μl. The viability of cells was above 80%, as confirmed with 0.2% (w/v) Trypan Blue staining (Countess II). Cells were captured in droplets. Following reverse transcription and cell barcoding in droplets, emulsions were broken and cDNA was purified using Dynabeads MyOne SILANE followed by PCR amplification per manual instruction. Between 15,000 to 25,000 cells were targeted for each sample. Samples were multiplexed together on one lane of 10x Chromium following the cell hashing protocol^74^. Final libraries were sequenced on the Illumina NovaSeq S4 platform (R1, 28 cycles; i7, 8 cycles; and R2, 90 cycles). The cell–gene count matrix was constructed using the Sequence Quality Control (SEQC) package^75^.

#### Data pre-processing

FASTQ files were generated from three different samples (ΔL, ΔS, anti−IFNAR1 ΔS) with three mice pooled together per condition. These files were then processed using the SEQC pipeline^75^ using the default parameters for a 10x single-cell 3′ library. This pipeline begins with aligning the reads against the provided mouse mm10 reference genome and resolving multi-mapping incidents. SEQC then corrects for unique molecular identifiers and cell barcodes and filters cells with high mitochondrial fraction (>20%), low library complexity (few unique genes expressed) and empty droplets. The resulting count matrix (cell × gene) was generated for each condition as the raw expression matrices.

Because each mouse was barcoded with a unique hashtag oligonucleotide for each sample, to demultiplex the cells, an in-house method known as SHARP (https://github.com/hisplan/sharp) was employed. Labels were assigned to either identify a cell as belonging to a specific mouse or as a doublet/low-quality droplet. The labeled cell barcodes and gene expression matrix were then concatenated together into one count matrix. Most of the downstream analysis and processing was conducted using the Scanpy software^76^.

#### Data cleanup

We began by filtering for lowly expressed genes, defined as those expressed in fewer than 32 cells in the combined dataset. The resulting count matrix was then normalized by library size (defined as the total RNA counts per cell), scaled by median library size and log_2_-transformed with a pseudocount of 0.1 for the combined dataset. For downstream analysis, we first performed dimensionality reduction using PCA to obtain the top 30 principal components, chosen based on the decay of associated eigenvalues, computed on the top 4,000 highly variable genes (HVGs). We then computed a *k*-nearest-neighbor graph representation of the cells on the obtained principal components (n_neighbors = 30). We visualized the cells on a two-dimensional projection using UMAP^77^ based on the implementation in Scanpy (using min_dist = 0.1 parameter). All cells from different samples were observed to group together based on their cell type, which indicated that no batch effect was present in the data (Fig. 3a). The cells were then clustered using PhenoGraph^78^ on the PCA space with *k* = 30. We ensured that the clusters were robust to variations around the chosen parameter of *k*. We measured consistency using adjusted rand index (as implemented in the Sklearn package in Python) and observed a high degree of consistency for values of *k* around 30. Upon close inspection of the obtained clusters, we observed one cluster that had low CD45 (PTPRC^−^) and high KRT8^+^ expression and two other clusters that had low CD45 and high expression of mitochondrial genes. Therefore, we decided to remove these clusters from further analysis.

#### IFN response signature

We first sought to broadly understand, on a per-cell-type basis, the response to IFN activity. We reasoned that to answer this, we ought to identify the genes that are most differential between anti-IFNAR1 and control ΔS. Therefore, we constructed an IFN signature by identifying the top 100 differentially upregulated genes in ΔS compared to anti-IFNAR1. The differential genes were identified using MAST^79^ and the top 100 genes were averaged on a per cell basis and plotted on the UMAP (Fig. 3c). Once the signature was constructed, we removed cells from the anti-IFNAR1 condition from further analysis to directly contrast ΔS and ΔL.

#### Analysis on ΔS and ΔL samples

The count matrix of CD45^+^ cells from the ΔS and ΔL samples included 15,334 cells (7,774 belonging to ΔS and 7,560 to ΔL) and 15,329 genes. To ensure that the observed heterogeneity was not impacted by these cell clusters, we re-processed the data using the same parameters as described above. Broad cell types were assigned to these clusters according to the average expression of known markers.

#### CD8^+^ T cells

We isolated cells identified as CD8^+^ T cells to analyze them separately. For this, the 6,080 CD8^+^ T cells were sub-clustered using PhenoGraph on top of the first 30 principal components (*k* = 30) using 1,500 HVGs. Using known markers, these PhenoGraph clusters were then annotated into further subtypes of CD8^+^ T cells based on the average expression of the markers in each subcluster.

#### Milo analysis on CD8^+^ T cells

We employed Milo^42^ to statistically quantify the changes in abundance of ΔS and ΔL specific cells among the CD8^+^ T-cell subtypes. Milo utilizes nearest-neighbor graphs to construct local neighborhoods (possibly overlapping) of cells and calculates and visualizes differential abundance of cells from different conditions in the obtained neighborhoods. For this analysis, we first constructed a *k*-nearest-neighbor graph (*k* = 30) on the first 30 principal components using the buildGraph function in Milo. Neighborhoods were calculated using the makeNhoods function (prop = 0.1, refined = TRUE). We used default parameters for countCells, testNhoods and calcNhoodDistance to calculate statistical significance and spatial false discovery rate correction and plotNhoodGraphDA (α = 0.5) to visualize the results. The color scale of the logFC uses blue to represent higher abundance of ΔL cells and red to represent higher abundance of ΔS cells and the size of the circle is proportional the number of cells belonging to the neighborhood. We further assigned each neighborhood a cell-type identity if more than 80% of the cells in a neighborhood belonged to a specific CD8^+^ T subtype, otherwise they were categorized as mixed.

#### Dendritic cells

Cells annotated as dendritic cells were isolated for further analysis. The 1,134 cells were clustered using PhenoGraph on top 30 principal components (*k* = 30) using 1,500 HVGs. The dendritic cells were further cell typed according to markers from previous studies^80^. The proportion of cells that belong to ΔL and ΔS in each cluster was calculated and plotted.

#### Macrophages

Cells labeled as macrophages (1,788 cells) were isolated. The cells were clustered using PhenoGraph on the top 30 principal components (*k* = 30) using 1,500 HVGs. These clusters were analyzed and annotated according to macrophage subtypes based on the DEGs computed in each cluster compared to the rest of the data using MAST. The proportion of cells that belong to ΔL and ΔS in each cluster was calculated and plotted.

#### B cells

Overall, 1,204 cells annotated as B cells were selected. The cells were clustered using PhenoGraph on the top 30 principal components (*k* = 30) using 1,500 HVGs. We obtained DEGs in each B-cell subcluster using MAST and utilized the results to distinguish distinct populations. The proportion of cells that belong to ΔL and ΔS in each cluster was calculated and plotted.

### Reporting summary

Further information on research design is available in the Nature Research Reporting Summary linked to this article.

### Supplementary information

Supplementary Information Supplementary Fig. 1

Reporting Summary

Supplementary Tables 1–5

### Source data

Source Data Fig. 1 Data from Fig. 1.

Source Data Fig. 2 Data from Fig. 2.

Source Data Fig. 3 Data from Fig. 3.

Source Data Fig. 4 Data from Fig. 4.

Source Data Fig. 5 Data from Fig. 5.

Source Data Fig. 6 Data from Fig. 6.

Source Data Fig. 7 Data from Fig. 7.

Source Data Fig. 8 Data from Fig. 8.

Source Data Extended Data Fig. 2 Data from Extended Fig. 2.

Source Data Extended Data Fig. 3 Data from Extended Fig. 3.

Source Data Extended Data Fig. 4 Data from Extended Fig. 4.

Source Data Extended Data Fig. 5 Data from Extended Fig. 5.

Source Data Extended Data Fig. 6 Data from Extended Fig. 6.

Source Data Extended Data Fig. 8 Data from Extended Fig. 8.

Source Data Extended Data Fig. 9 Data from Extended Fig. 9.

Source Data Extended Data Fig. 10 Data from Extended Fig. 10.

Source Data Fig. 1 Uncropped gels from Fig. 1.

Source Data Fig. 2 Uncropped gels from Fig. 2.

Source Data Fig. 5 Uncropped gels from Fig. 5.

Source Data Extended Data Fig. 1 Uncropped gels from Extended Data Fig. 1.

Source Data Extended Data Fig. 2 Uncropped gels from Extended Data Fig. 2.

## Data Availability

All datasets have been deposited and made publicly available: sparse whole-genome sequencing (accession PRJNA866212), bulk RNA-seq (GSE210953), scRNA-seq (GSE210818). Previously published data that were re-analyzed are available from EGA under accession code EGAS00001002543^43,44^. Genomic data from the TCGA Research Network (http://cancergenome.nih.gov/) cohort was analyzed in cBioPortal (https://www.cbioportal.org/). Source data are provided for this study and all other data supporting the findings are available from the corresponding author upon reasonable request. Source data are provided with this paper.

## Code availability

No unique code was developed for this study.
