# Supplementary material for: MACHETE identifies interferon-encompassing chromosome 9p21.3 deletions as mediators of immune evasion and metastasis
Source: Nat Cancer. 2022 Nov 7;3(11):1367–85. doi: 10.1038/s43018-022-00443-5 (PMC9701143; doi:10.1038/s43018-022-00443-5)

nature cancer

Article https://doi.org/10.1038/s43018-022-00443-5

MACHETE identifies interferon-encompassing chromosome 9p21.3 deletions as mediators of immune evasion and metastasis

In the format provided by the authors and unedited

Supplementary Figure 1

Lymphoid panel

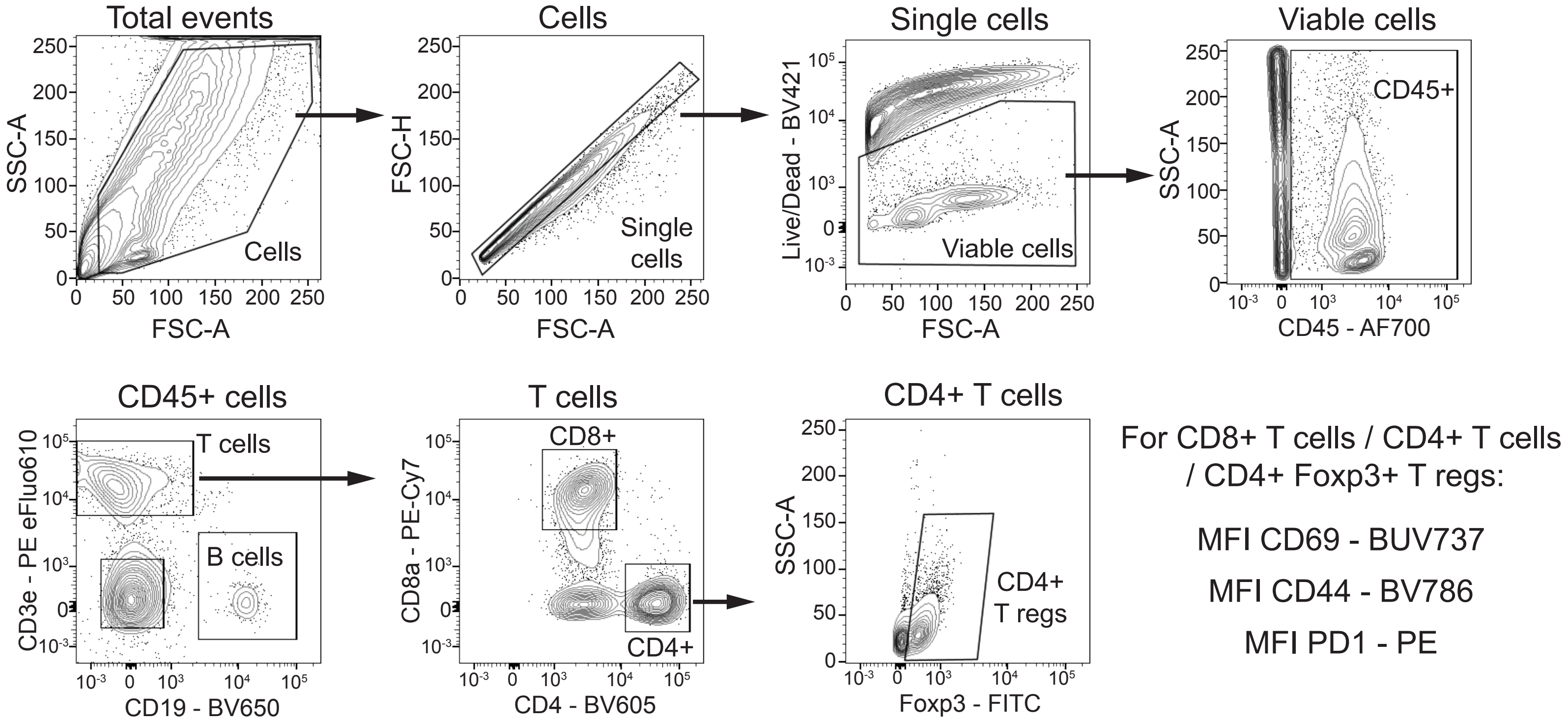

Myeloid panel

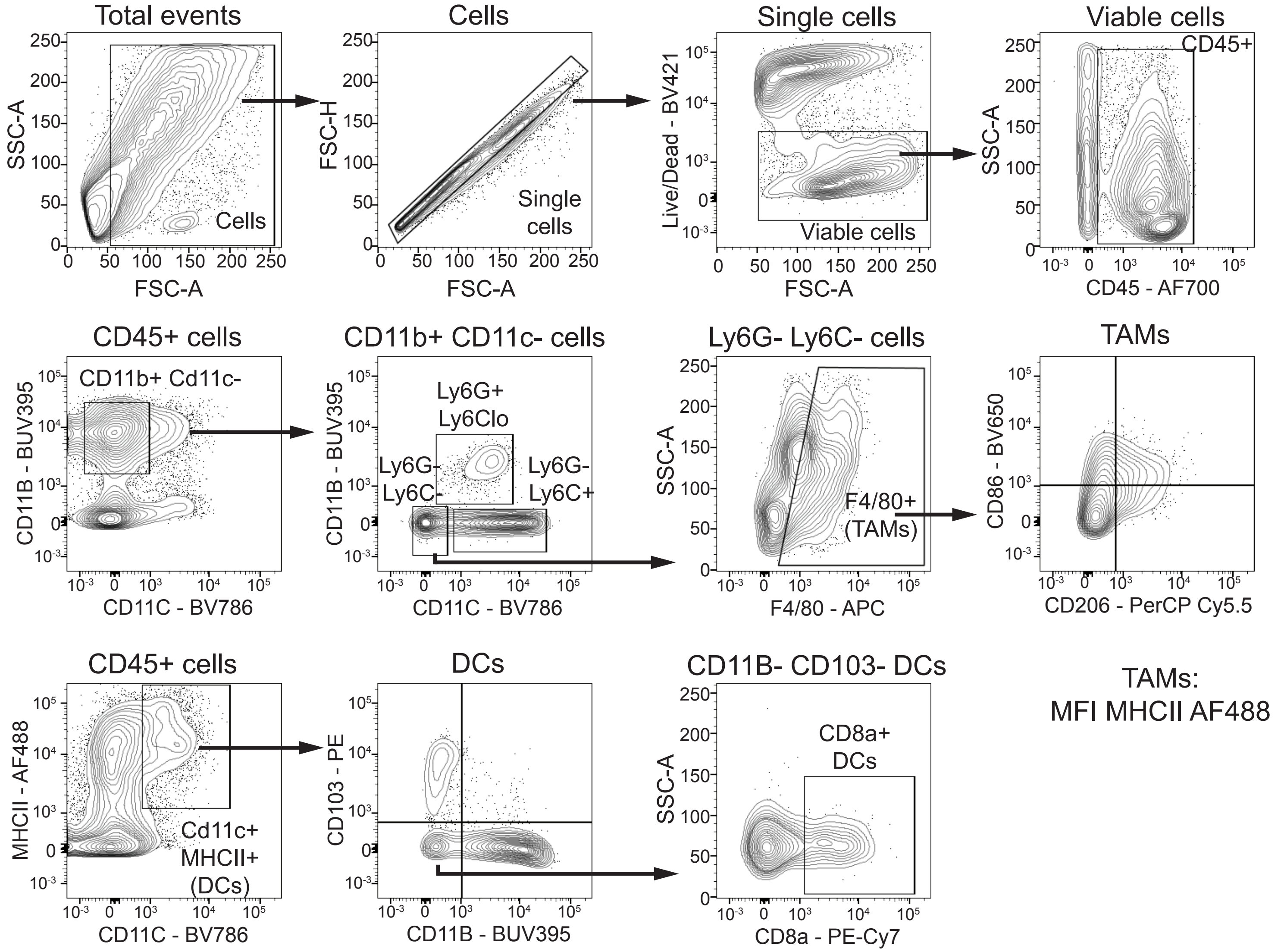

Supplement: Supplementary file 1 — Supplementary Fig. 1 [file 43018_2022_443_MOESM1_ESM.pdf]
