# Supplementary material for: MACHETE identifies interferon-encompassing chromosome 9p21.3 deletions as mediators of immune evasion and metastasis
Source: Nat Cancer. 2022 Nov 7;3(11):1367–85. doi: 10.1038/s43018-022-00443-5 (PMC9701143; doi:10.1038/s43018-022-00443-5)
Supplement: Supplementary file 2 — Reporting Summary [file 43018_2022_443_MOESM2_ESM.pdf]

nature portfolio

Corresponding author(s): Scott W. Lowe, PhD

Last updated by author(s): Sep 8, 2022

Reporting Summary

Nature Portfolio wishes to improve the reproducibility of the work that we publish. This form provides structure for consistency and transparency in reporting. For further information on Nature Portfolio policies, see our Editorial Policies and the Editorial Policy Checklist.

Statistics

For all statistical analyses, confirm that the following items are present in the figure legend, table legend, main text, or Methods section.

n/a	Confirmed	
☐	☒	The exact sample size (*n*) for each experimental group/condition, given as a discrete number and unit of measurement
☐	☒	A statement on whether measurements were taken from distinct samples or whether the same sample was measured repeatedly
☐	☒	The statistical test(s) used AND whether they are one- or two-sided<br>*Only common tests should be described solely by name; describe more complex techniques in the Methods section.*
☒	☐	A description of all covariates tested
☐	☒	A description of any assumptions or corrections, such as tests of normality and adjustment for multiple comparisons
☐	☒	A full description of the statistical parameters including central tendency (e.g. means) or other basic estimates (e.g. regression coefficient) AND variation (e.g. standard deviation) or associated estimates of uncertainty (e.g. confidence intervals)
☐	☒	For null hypothesis testing, the test statistic (e.g. *F*, *t*, *r*) with confidence intervals, effect sizes, degrees of freedom and *P* value noted<br>*Give P values as exact values whenever suitable.*
☒	☐	For Bayesian analysis, information on the choice of priors and Markov chain Monte Carlo settings
☒	☐	For hierarchical and complex designs, identification of the appropriate level for tests and full reporting of outcomes
☒	☐	Estimates of effect sizes (e.g. Cohen's *d*, Pearson's *r*), indicating how they were calculated

Our web collection on statistics for biologists contains articles on many of the points above.

Software and code

Policy information about availability of computer code

Data collection	NGS data presented in the manuscript was collected with standard approaches and is described in the Methods section of the manuscript.
Data analysis	Genomic and transcriptional Ddata analysis was done with standard approaches (DE Seq2, GSEA analysis) and are described in the Methods section of the manuscript. Flow cytometry data was analyzed with FlowJo V10.0 and BD FACS Diva

For manuscripts utilizing custom algorithms or software that are central to the research but not yet described in published literature, software must be made available to editors and reviewers. We strongly encourage code deposition in a community repository (e.g. GitHub). See the Nature Portfolio guidelines for submitting code & software for further information.

Data

Policy information about availability of data

All manuscripts must include a data availability statement. This statement should provide the following information, where applicable:

- Accession codes, unique identifiers, or web links for publicly available datasets
- A description of any restrictions on data availability
- For clinical datasets or third party data, please ensure that the statement adheres to our policy

All data are uploaded to the GEO database with the following accession codes: Bulk-RNA - GSE210593; scRNA - GSE210818; sWGS - PRJNA866212

Human research participants

Policy information about studies involving human research participants and Sex and Gender in Research.

Reporting on sex and gender	N/A
Population characteristics	N/A
Recruitment	N/A
Ethics oversight	N/A

Note that full information on the approval of the study protocol must also be provided in the manuscript.

Field-specific reporting

Please select the one below that is the best fit for your research. If you are not sure, read the appropriate sections before making your selection.

☒ Life sciences ☐ Behavioural & social sciences ☐ Ecological, evolutionary & environmental sciences

For a reference copy of the document with all sections, see nature.com/documents/nr-reporting-summary-flat.pdf

Life sciences study design

All studies must disclose on these points even when the disclosure is negative.

Sample size	We determined at least 8-10 mice per experimental group in in tumor initiation and metastasis experiments. These numbers are based on a type I error of 5% (a = 0.05) and power of 80% (type II error, b = 0.8).
Data exclusions	No data was excluded from the analysis.
Replication	All deletions analyzed were generated with two independent sets of sgRNAs to rule out possible off-target effects and these conditions were treated as equivalent. Replicates were successful and behaved consistently. Experiments were repeated in independent cohorts of mice with at least 5 independent mice per condition. For secondary transplants 2-4 independent cell lines of each genotype were used.
Randomization	Mice were randomized for treatments cohorts (Figure 5 and ED Figure 4) onnce engrafted tumors reached a diameter of 5 mm (PDEC) or 100 mm3 (B16F10). No differences in size were observed at the onset of treatments.
Blinding	Blinding was used in metastasis scoring. For remaining experiments no blinding was used.

Reporting for specific materials, systems and methods

We require information from authors about some types of materials, experimental systems and methods used in many studies. Here, indicate whether each material, system or method listed is relevant to your study. If you are not sure if a list item applies to your research, read the appropriate section before selecting a response.

Materials & experimental systems

n/a	Involved in the study	
☐	☒	Antibodies
☐	☒	Eukaryotic cell lines
☒	☐	Palaeontology and archaeology
☐	☒	Animals and other organisms
☒	☐	Clinical data
☒	☐	Dual use research of concern

Methods

n/a	Involved in the study	
☒	☐	ChIP-seq
☐	☒	Flow cytometry
☒	☐	MRI-based neuroimaging

Antibodies

Antibodies used	Blocking/Depleting antibodies<br>IgG Control, MOPC21 clone, BioXCell<br>anti-IFNAR1, MAR15A3, BioXCell<br>anti-CD8a, Clone 2.43, BioXCell

anti-CD4, Clone GK1.5, BioXCell
anti-CD20, Clone SA271G2, BioLegend

Flow Cytometry Panels

Lymphoid

Marker Fluorophore Clone Company Concentration
CD45 AF700 30-F11 BioLegend 1/400
CX3CR1 BV510 SA011F11 BioLegend 1/400
CD3 PE Fluo610 145-2C11 eBioscience 1/100
CD4 BV605 RM4 5 BD 1/200
CD8 PE Cy7 53-6.7 BioLegend 1/400
PD-L1 APC Cy7 10F.9G2 BioLegend 1/400
CD44 BV786 IM7 BioLegend 1/400
CD69 BUV737 H1.2F3 BD 1/400
CD19 BV650 1D3 BD 1/400
PD1 PE 29F.1A12 BioLegend 1/400
CD62L APC Cy7 MEL-14 BioLegend 1/400
TCR gd BUV395 V65 BD 1/200
Foxp3 FITC FJK-16s eBioscience 1/100
Viability BV421 Live/Dead Invitrogen 1/1000
Fc Block NA 2.4G2 BD 1/200

Myeloid

Marker Fluorophore Clone Company Concentration
CD45 AF700 30-F11 BioLegend 1/400
CD11b BUV395 M1/70 BD 1/800
CD86 BV650 GL-1 BioLegend 1/400
Ly6C APC Cy7 AL-21 BD 1/400
Ly6G BV605 1A8 BD 1/400
CD11c BV786 N418 BioLegend 1/800
CD206 PerCP Cy5.5 C068C2 BioLegend 1/400
F4/80 APC BM8 BioLegend 1/400
CD103 PE-Cy7 2E7 BioLegend 1/400
CX3CR1 BV510 SA011F11 BioLegend 1/400
CD8 PE Cy7 53-6.7 BioLegend 1/400
Viability BV421 Live/Dead Invitrogen 1/1000

Fc Block NA 2.4G2 BD 1/200

Validation

All antibodies used are commercially available and validated across multiple other publications. All gating was determined with fluorescence minus one (FMO) controls.

Eukaryotic cell lines

Policy information about cell lines and Sex and Gender in Research

Cell line source(s)

ATCC (NIH3T3, B16F10); Laboratory of Dr. Dafna Bar-Sagi (Pancreatic Ductal Epithelial Cells)

Authentication

PDEC cells were analyzed by genome wide SNP analysis to verify their background (female C57BL/6n). Cells were then genotyped and functionally validated to show the presence of the driver mutations (KrasG12D, and CRISPR-Cas9 inactivation of Trp53). Further validation was done by transcriptional profiling via RNA Seq and low pass whole genome sequencing of tumor-derived cells. All these analysis confirmed the origin and genotype of the cells used in the study. ATCC derived cell lines were not independently authenticated.

Mycoplasma contamination

Cells tested negative for mycoplasma.

Commonly misidentified lines
(See ICLAC register)

None of these lines were used in this study.

Animals and other research organisms

Policy information about studies involving animals; ARRIVE guidelines recommended for reporting animal research, and Sex and Gender in Research

Laboratory animals

Mice were kept in day/night cycles of 12 hours with controlled temperature, humidity, and fed ad libitum.
Mus musculus; C57BL/6n; females; 6-8 weeks
Mus musculus; Foxn1 Nu (Nude); females; 6-8 weeks
Mus musculus; NOD/SCID Il2rg-/- (NSG) females; 6-8 weeks

Wild animals

No wild animals were used.

Reporting on sex	Experiments were done with female host mice to match the sex of the engineered PDEC cell lines and avoid potential immune recognition.
Field-collected samples	No field work in this study.
Ethics oversight	All mouse work was approved by the MSKCC IACUC under protocol number 11-06-018. Maximum tumor burden was established following IRB guidelines: when a tumor reached 10% of weight (PDEC models), reached 1500 mm3 (B16F10 models), or mice had overt disease or signs of distress. All mice reaching any of these endpoint criteria were euthanized.

Note that full information on the approval of the study protocol must also be provided in the manuscript.

Flow Cytometry

Plots

Confirm that:

☒ The axis labels state the marker and fluorochrome used (e.g. CD4-FITC).

☒ The axis scales are clearly visible. Include numbers along axes only for bottom left plot of group (a 'group' is an analysis of identical markers).

☒ All plots are contour plots with outliers or pseudocolor plots.

☒ A numerical value for number of cells or percentage (with statistics) is provided.

Methodology

Sample preparation	Tumor fragments were then transferred to a solution of type V collagenase (Sigma C9263, 1 mg/mL in 1X HBSS) supplemented with soy trypsin inhibitor (Gibco, 0.1 mg/mL) and DNAse I (Sigma, 0.1 mg/mL). Tumor pieces in this disaggregation buffer were transferred to a GentleMACS tube and loaded into the OctoDissociator (Miltenyi). Samples were treated with the mTDK1 program, after which 5 mL of FACS Buffer (PBS 1X, 2% FBS) was added to the sample and the mix was filtered through a 100 m mesh (BD). The resulting cell suspension was centrifuged and resuspended in FACS buffer. Cells were then treated with Fc block (BD, 1:200 dilution) and incubated at 4C for 15 minutes. Cells were then stained with anti-CD45 AF700 (BD, 1:400 dilution) for 30 min at 4C. Cells were then washed and resuspended in FACS buffer supplemented with DAPI (Sigma, 1 g/mL final). Stained cell suspensions were then analyzed in a MA900 sorter (Sony). EGFP+ cells were analyzed within the CD45-, DAPI- population.<br>For multi-parametric flow cytometry analysis, tumor cell suspensions were generated as above, and cells were stained with LIVE/DEAD fixable viability dye (Invitrogen) for 30 min at 4C. After this, cells were washed, incubated with Fc block (BD, 1:200) for 15 min at 4 C, and then stained with conjugated antibody cocktails (see Table S5 for antibody panels) for 30 min at 4C. After staining cells were washed and fixed (BD Cytofix) for 20 min at 4C, washed again, and stored for analysis. Samples were analyzed in a BD LSRFortessa with 5 lasers, where gates were set by use of fluorescence-minus-one (FMO) controls.
Instrument	LSR Fortessa
Software	Data acquisition was done with BD FACS Diva software. Data analysis was done in FlowJo v10.0.
Cell population abundance	Only analysis was performed.
Gating strategy	Tumor and infiltrating immune cells were identified (FSC/SSC), and single cell events were selected (FSC-A/FSC-H). Live cells were identified (DAPI or LIVE/DEAD / FSC) and cells were then gated for CD45+. For lympoid-specific panels CD45+ were subdivided in T and B cell subsets (CD3e or CD19). Within CD3e, cells were then further divided in gd T/CD4/CD8 subsets, and activation markers were assessed (CD44, CD69, PD-1, CD62L, CX3CR1). For myeloid panels, CD45+ cells were gated in CD11b+ or Cd11c+ subsets. Cd11b+ cells were analyzed for Ly6G and Ly6C to identify granulocytic or monocytic myeloid derived suppressor cells or tumor associated macrophages (TAMs). TAMs were then gated for F4/80 expression and analyzed for CD86 or CD206 to establish their polarization to M1 or M2 respectively. Within Cd11c+ subsets, cells were gated based on CD103, Cd11b, or CD8 to define abundance of dendritic cell subsets.

☒ Tick this box to confirm that a figure exemplifying the gating strategy is provided in the Supplementary Information.
