# Supplementary figures and images for: MACHETE identifies interferon-encompassing chromosome 9p21.3 deletions as mediators of immune evasion and metastasis
Source: Nat Cancer. 2022 Nov 7;3(11):1367–85. doi: 10.1038/s43018-022-00443-5 (PMC9701143; doi:10.1038/s43018-022-00443-5)

Figure 1

Figure 1D  
KI PCR

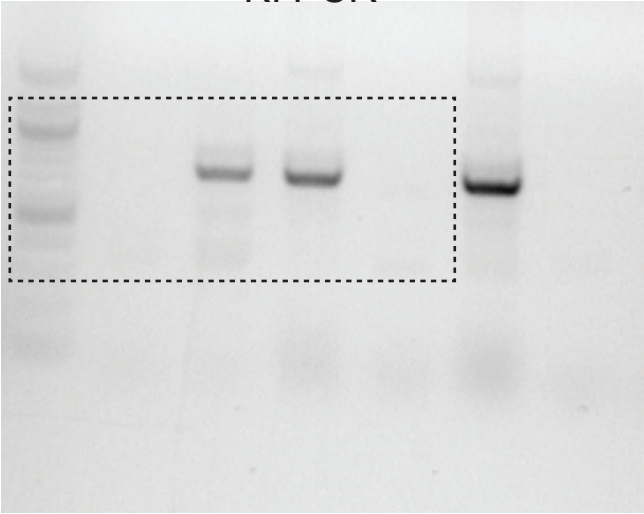

Figure 1D  
 $\Delta 11B3$  PCR

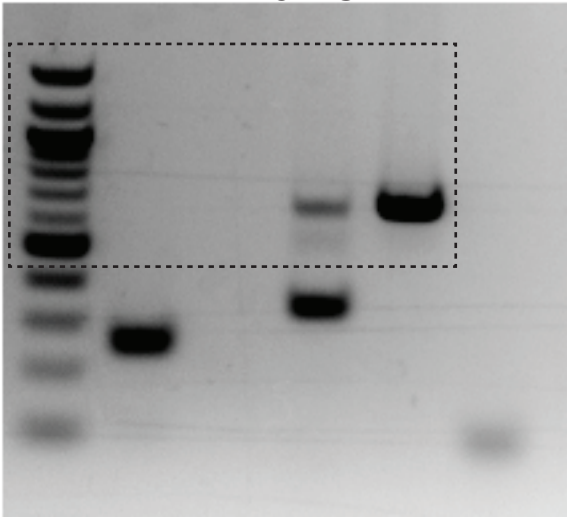

Figure 1I  
Cassette PCR

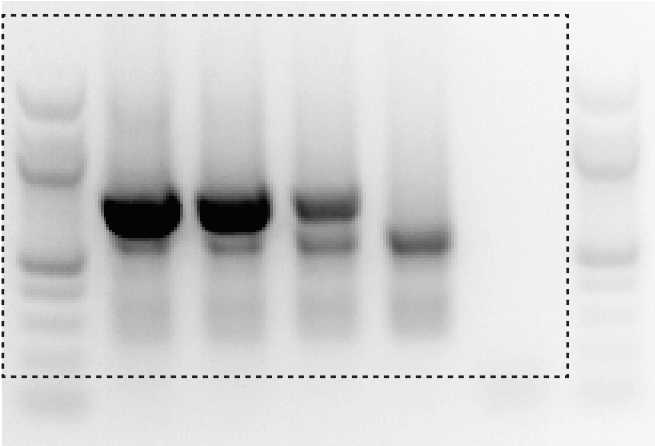

Figure 1I  
 $\Delta 7q11-22$  PCR

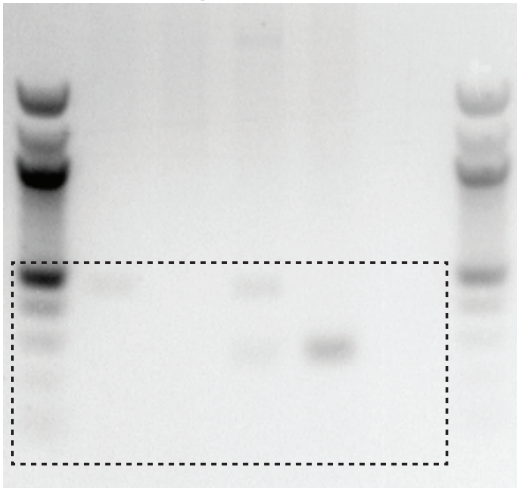

Supplement: Supplementary file 20 — Uncropped gels from Fig. 1. [file 43018_2022_443_MOESM20_ESM.pdf]

Figure 2

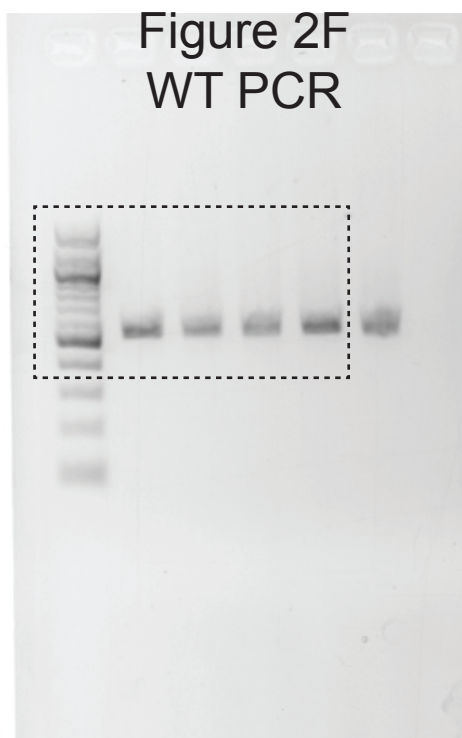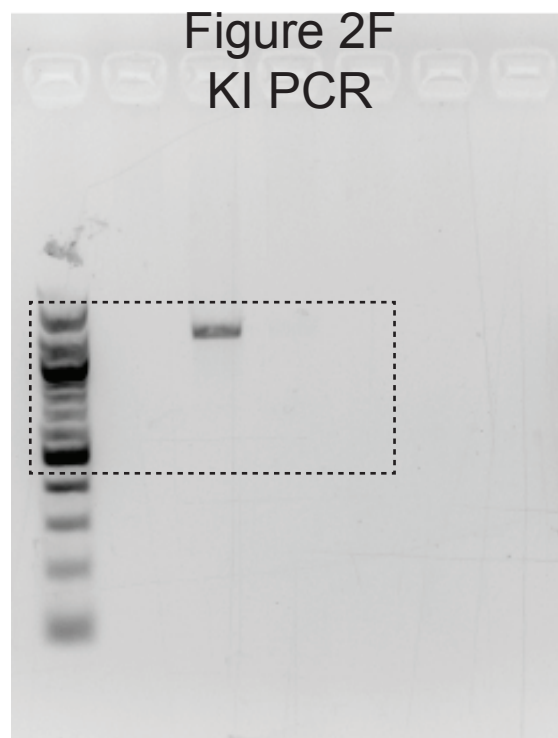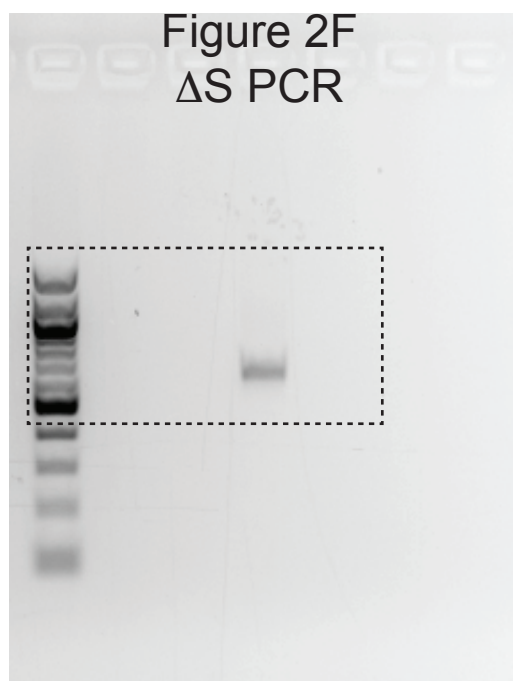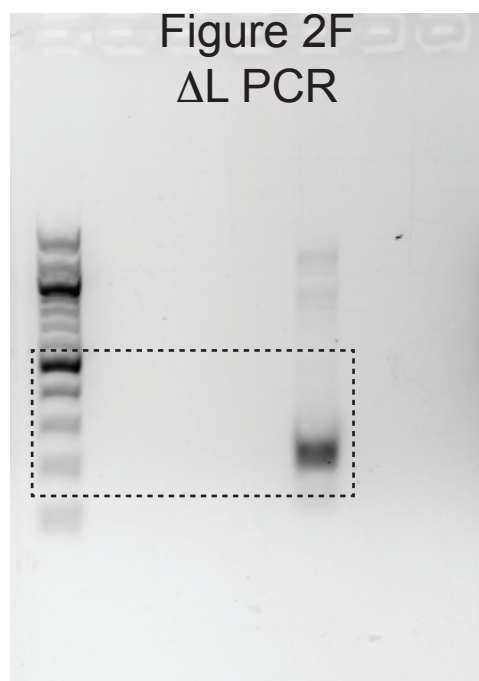

Supplement: Supplementary file 21 — Uncropped gels from Fig. 2. [file 43018_2022_443_MOESM21_ESM.pdf]

Figure 5

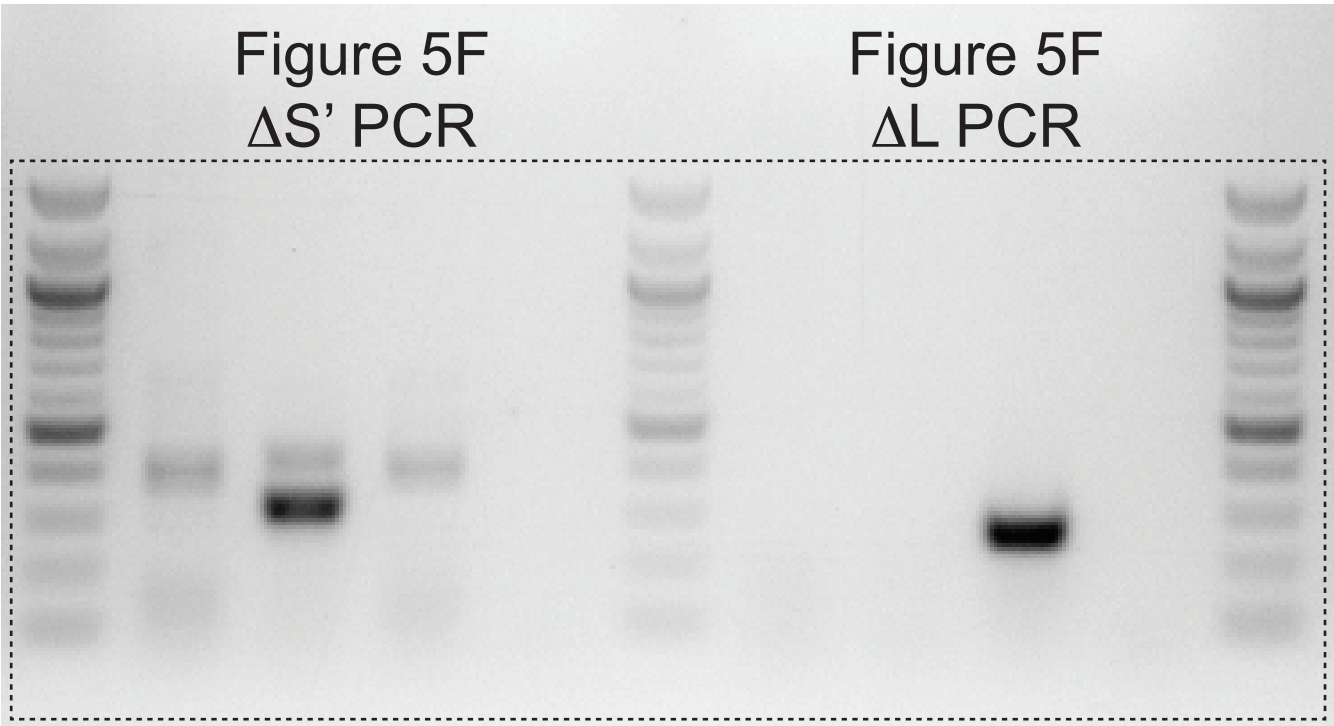

Supplement: Supplementary file 22 — Uncropped gels from Fig. 5. [file 43018_2022_443_MOESM22_ESM.pdf]

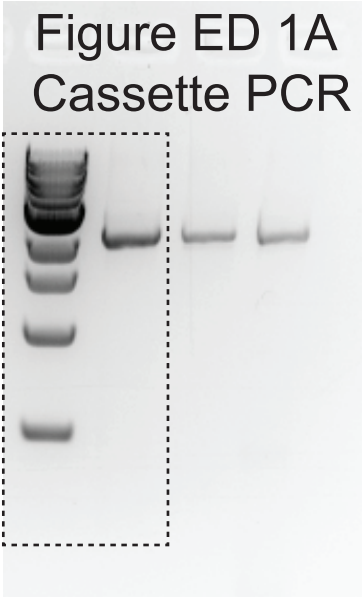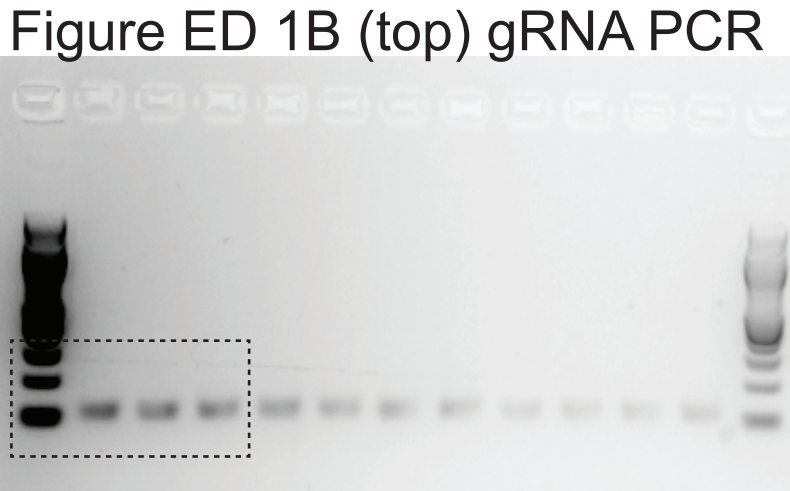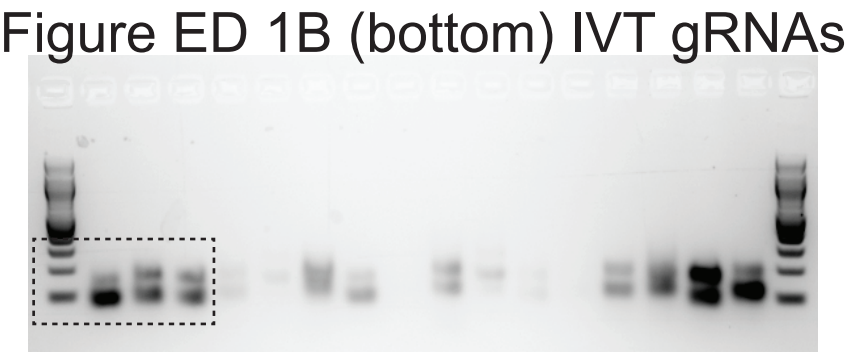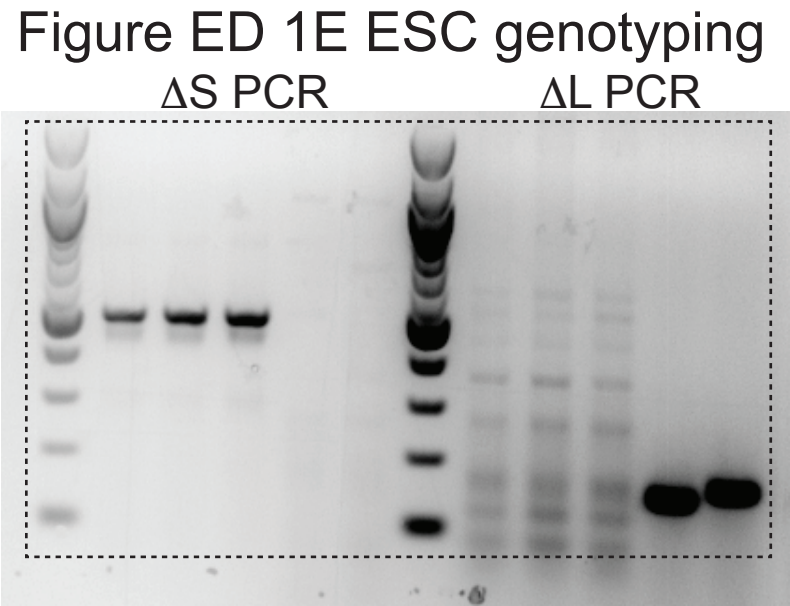

Supplement: Supplementary file 23 — Uncropped gels from Extended Data Fig. 1. [file 43018_2022_443_MOESM23_ESM.pdf]

Figure ED 2G  
 $\Delta$ L-A PCR

Figure ED 2G  
 $\Delta$ L-B PCR

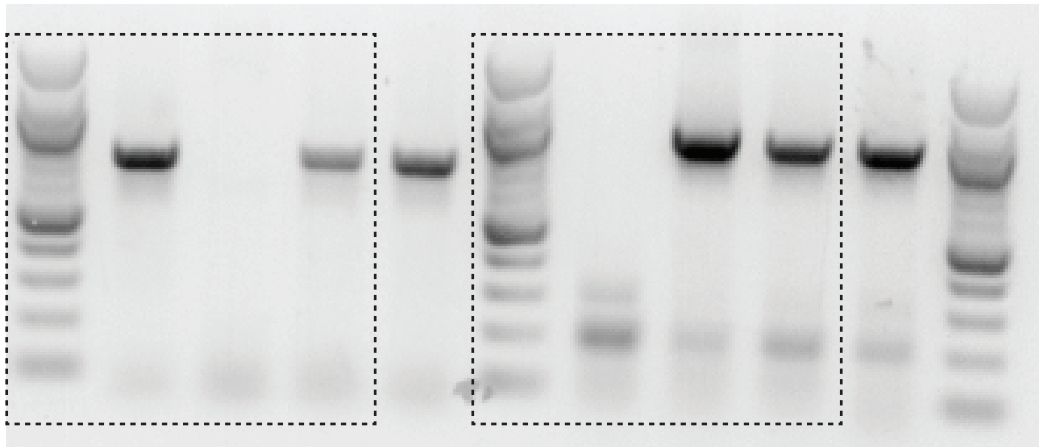

Supplement: Supplementary file 24 — Uncropped gels from Extended Data Fig. 2. [file 43018_2022_443_MOESM24_ESM.pdf]
